# Microdissection testicular sperm extraction outcomes in azoospermic patients post-orchidopexy surgery: A systematic review and meta-analysis

**DOI:** 10.1371/journal.pone.0313866

**Published:** 2024-11-15

**Authors:** Hao-nan He, Hong Xiao, Rui-jie Yao, Shi-jie Liao, Jun-hang Zheng, Hui-liang Zhou

**Affiliations:** Department of Andrology and Sexual Medicine, First Affiliated Hospital of Fujian Medical University, Fuzhou, China; University of Tehran, ISLAMIC REPUBLIC OF IRAN

## Abstract

Cryptorchidism is a common cause of male infertility, often necessitating microdissection testicular sperm extraction (m-TESE) for sperm retrieval post-surgery. However, uncertainties persist regarding m-TESE outcomes and influencing factors following cryptorchidism surgery. A systematic review and meta-analysis were conducted to evaluate sperm retrieval rates (SRR) among patients undergoing m-TESE after cryptorchidism surgery. Factors including age at orchidopexy, age at m-TESE, type of cryptorchidism, serum hormone levels, testicular volume, and interval from surgery to m-TESE were analyzed for their impact on SRR.Nine studies encompassing 935 patients were included. The overall SRR was 57% (95% confidence interval [CI] 51% to 63%). Compared to patients with negative sperm retrieval (SR-), patients with positive sperm retrieval (SR+) underwent m-TESE at an older age (1.81 years; 95% CI 1.17 to 2.45) and orchidopexy at a younger age (-3.35 years; 95% CI -6.34 to -0.36). Different types of cryptorchidism (including high scrotal, inguinal canal, intra-abdominal) significantly influenced SRR (P<0.05). Serum testosterone, follicle-stimulating hormone, luteinizing hormone levels and testicular volume showed no significant correlation with SRR (P>0.05). Furthermore, SR- patients typically experienced shorter intervals from orchidopexy to m-TESE compared to SR+ patients (34.09 months; 95% CI 0.40 to 67.77). Earlier orchidopexy and much later m-TESE procedures, as well as undescended testis closer to the scrotum, increase the likelihood of successful sperm retrieval. Orchidopexy for cryptorchidism should be done as early as possible, whether it is performed before 18 months of age or detected at a much older age. In patients with undetected cryptorchidism and azoospermia after puberty, m-TESE should not be performed immediately after orchidopexy, the optimal interval from orchidopexy to m-TESE still requires further study.

## Introduction

Cryptorchidism is one of the most common congenital anomalies in male neonates, with a prevalence about 1%-4% in term infants and higher in preterm infants [1–3]. Cryptorchidism is associated with a higher risk of testicular germ cell tumors and impaired spermatogenesis, which can result in non-obstructive azoospermia (NOA) and contribute to male infertility [4]. Previous studies have shown that cryptorchidism accounts for 17.2% of patients with non-obstructive azoospermia [5]. Therefore, according to the European Association of Urology (EAU) guidelines, orchidopexy should be performed before 12 months or 18 months at the latest for optimal fertility. Despite undergoing orchidopexy, some individuals may still face fertility challenges due to persistent spermatogenic dysfunction, leading to non-obstructive azoospermia (NOA). It is widely accepted that the testes of NOA patients may contain spermatozoa, and therefore the implementation of in vitro fertilization is beneficial for these patients to obtain biological offspring [6]. In previous studies in patients with NOA, microdissected testicular sperm extraction (m-TESE) was shown to be a newer sperm retrieval technique with a higher sperm retrieval rate (SRR) compared to others, e.g.testicular sperm aspiration (TESA) and conventional testicular sperm extraction (c-TESE), this indicates that patients with cryptorchidism who undergo m-TESE have a high probability of achieving biological offspring. In this method, the testes are placed under a 20x surgical microscope, the surgeon selects swollen seminiferous tubules, and then takes tissue samples from the tubules to find sperm. This technique is able to finely distinguish the testicular tissue most likely to contain sperm, and reduces damage to the testicular vasculature, and is considered a better sperm retrieval technique [7]. Nevertheless, the administration of m-TESE has the potential to further impair the spermatogenic function of the testis [8].

Recent studies have interpreted several predictors of sperm retrieval outcomes, such as age at orchidopexy, testicular volume, location of undescended testes, concentrations of testosterone, Follicle-Stimulating Hormone (FSH) and Luteinizing Hormone (LH) [9–17]. However, these studies were single-centre and had small sample sizes, and there was no strong evidence to support the association of these factors with m-TESE outcomes. To address this issue, we conducted a meta-analysis of relevant studies. Our objective is to investigate the relationship between multiple factors—including age at m-TESE, age at orchidopexy, interval from orchidopexy to m-TESE, undescended testes location, testicular volume, and levels of testosterone, FSH, and LH—and the success outcome of m-TESE in patients with NOA who have undergone orchidopexy for cryptorchidism. We hope that this study will find the best time to perform m-TESE and orchidopexy. For patients with extremely negative factors of m-TESE, we may suggest using artificial insemination with donor sperm rather than performing sperm extraction to reduce additional testicular damage.

## Materials and methods

### Literature search

We adhered to the Preferred Reporting Items for Systematic Reviews and Meta-Analyse (PRISMA) guidelines when conducting our study. The study protocol was published in PROS-PERO (registration number: CRD42024567368).

Two independent authors (Haonan He and Hong Xiao) searched PubMed, EMBASE, and Web of Science databases to obtain all relevant data up until July 2024. Only peer-reviewed and published in English articles will be included. On PubMed, we using the following search terms: ((sperm retrieval rates) OR (testicular sperm extraction) OR(micro dissection testicular sperm extraction) OR (Micro-TESE) OR(M-TESE)) AND ((Cryptorchidism) OR (Cryptorchism) OR (Testes, Undescended) OR(Undescended Testes) OR(Testis, Undescended) OR(cryptorchid) OR(Cryptorchidism, Unilateral Or Bilateral) OR(Undescended Testis) OR(Bilateral Cryptorchidism) OR(Cryptorchidism, Bilateral) OR(Unilateral Cryptorchidism) OR(Cryptorchidism, Unilateral) OR(Abdominal Cryptorchidism) OR(Cryptorchidism, Abdominal) OR(Inguinal Cryptorchidism) OR(Cryptorchidism, Inguinal) OR(orchidopexy)).Other databases search terms are presented in S5 File.

### Study selection and data extraction

The selected studies met the following inclusion criteria: (i) included participants diagnosed with NOA who underwent m-TESE and orchidopexy for cryptorchidism; (ii) controlled trials, cohort studies, case-control studies, cross-sectional surveys, and descriptive studies; (iii) original human studies; (iv)included sperm retrieval rate as an outcome measure; and (v) were published before July 2024.

The exclusion criteria were: (a) reviews, conference abstracts, animal experiments, and case reports with <10 cases; (b) absence of micro dissected testicular sperm extraction for sperm retrieval; and (c) inclusion of overlapping participants.

Two authors independently evaluated the search results, Haonan He and Hong Xiao, thoroughly assessed the articles to confirm their relevance to the study and ultimately determined which ones would be included. The search strategy was initially created for PubMed and then adjusted for the other databases. The following data were obtained from relevant articles: publication years, authors’ names, ages of the patients, ages at orchidopexy, interval from orchidopexy to m-TESE, location of undescended testes, testicular volume, concentrations of testosterone, FSH, LH, and sperm retrieval rate.

### Quality assessment

The Quality Assessment Tool for Before-After (Pre-Post) Studies with No Control Group developed by the National Institutes of Health (NIH) was used to evaluate the quality of the included uncontrolled before-after studies. The tool consists of 12 items that assess the risk of bias in six domains: selection bias, study design, confounders identification and adjustment, blinding, data collection methods, and withdrawals and dropouts. Each item can be rated as "yes," "no," "not reported," or "not applicable" based on the information provided in the study.

The overall quality of each study is then classified as "good", "fair" or "poor" depending on the number of "yes" responses. In our analysis, we applied the tool to evaluate the quality of studies that met our inclusion criteria. Specifically, we checked if the study had a clear description of the intervention and outcome measures, if the study population was well-defined and representative of the target population, if the data collection method was reliable and valid, if the study accounted for potential confounding factors, if the outcome assessment was valid and reliable, and if the study reported sufficient data to allow for a meta-analysis. However, in this analysis, all included studies had no group-level interventions, the 12th item must always be marked as "Not applicable," leading to a maximum potential quality score of 11.

Two reviewers (Haonan He and Hong Xiao) independently assessed the quality of the included studies using the tool. Any discrepancies were resolved by discussion and consensus.The results of the quality assessment are presented in Table 1.

**Table 1:** Summary of key characteristics from the 9 studies included in the meta-analysis.

Study	Design	Patient (n)	SRR(%)	Country	Predictive factors	Quality score
Cayan et al. [9] 2020	Retrospective Study	327	53.00	Türkiye	Age at orchidopexy, T, FSH	9
Sangster et al. [10] 2020	Retrospective Study	12	33.33	UK	Age at m-TESE, Unilateral/Bilateral, Location of undescended testes, T, FSH, Testicular volume, Interval from orchidopexy to m-TESE	7
Saber-Khalaf et al. [11] 2022	Retrospective Study	103	62.14	Egypt	Age at m-TESE, Age at orchidopexy, Bilateral, T, FSH, LH, Testicular volume, Interval from orchidopexy to m-TESE	9
Li et al. [12] 2020	Retrospective Study	18	77.78	China	Age at m-TESE, Age at orchidopexy, Unilateral/Bilateral, T, FSH, LH, Interval from orchidopexy to m-TESE	9
Ozan et al. [13] 2019	Retrospective Study	148	61.49	Türkiye	Age at m-TESE, Age at orchidopexy, Unilateral/Bilateral, T, FSH	9
Arasteh et al. [14] 2024	Retrospective Study	56	46.43	Iran	Age at m-TESE,Age at orchidopexy,Bilateral,Location of undescended testes,T,FSH,LH,Testicular volume	8
Chen et al. [15] 2022	Retrospective Study	162	48.15	China	Age at m-TESE,Age at orchidopexy,Unilateral/Bilateral,Location of undescended testes,T,FSH,LH,Testicular volume,Interval from orchidopexy to m-TESE	9
Xu et al.[16] 2023	Retrospective Study	73	68.5	China	Age at orchidopexy, Unilateral/Bilateral, T, FSH, LH, Testicular volume	8
Osaka et al.[17] 2020^a^	Retrospective Study	36	55.56	Japan	Age at m-TESE, Age at orchidopexy, T, LH, Testicular volume, Interval from orchidopexy to m-TESE	7

T: Testosterone;FSH:Follicle-Stimulating Hormone;LH:Luteinizing Hormone

^a^Data from the Osaka et al. study are presented as means (ranges) and could not be analyzed in subgroups.

### Statistical analysis

Excel 2020 (Microsoft, Redmond, USA) was used to extract date. Review Manager 5.3 (Cochrane Collaboration, Oxford, UK) was used to conduct the meta-analysis. The *I*^*2*^ statistic was used to assess the heterogeneity among the studies included in the analysis. *I*^*2*^>50.0% showed a high level of heterogeneity. In such cases, the random-effects model was used to analyze the combined results. To determine the stability of the outcomes, a sensitivity analysis was performed by systematically excluding individual studies. Alternatively, if the heterogeneity was low (*I*^*2*^ < 50.0%), a fixed-effects model was used. To assess the publication bias, Egger’s test and funnel plots were used. StataSE 16.0 (StataCorp, College Station, TX, USA) was used for Egger’s test and the sensitivity analysis. P < 0.05 was considered statistically significant. For data mentioned in the records that approximately follows a normal distribution, the method suggests approximating the median as the mean and estimating the standard deviation as the quartile range divided by (2×1.349).

## Results

**Fig 1:**
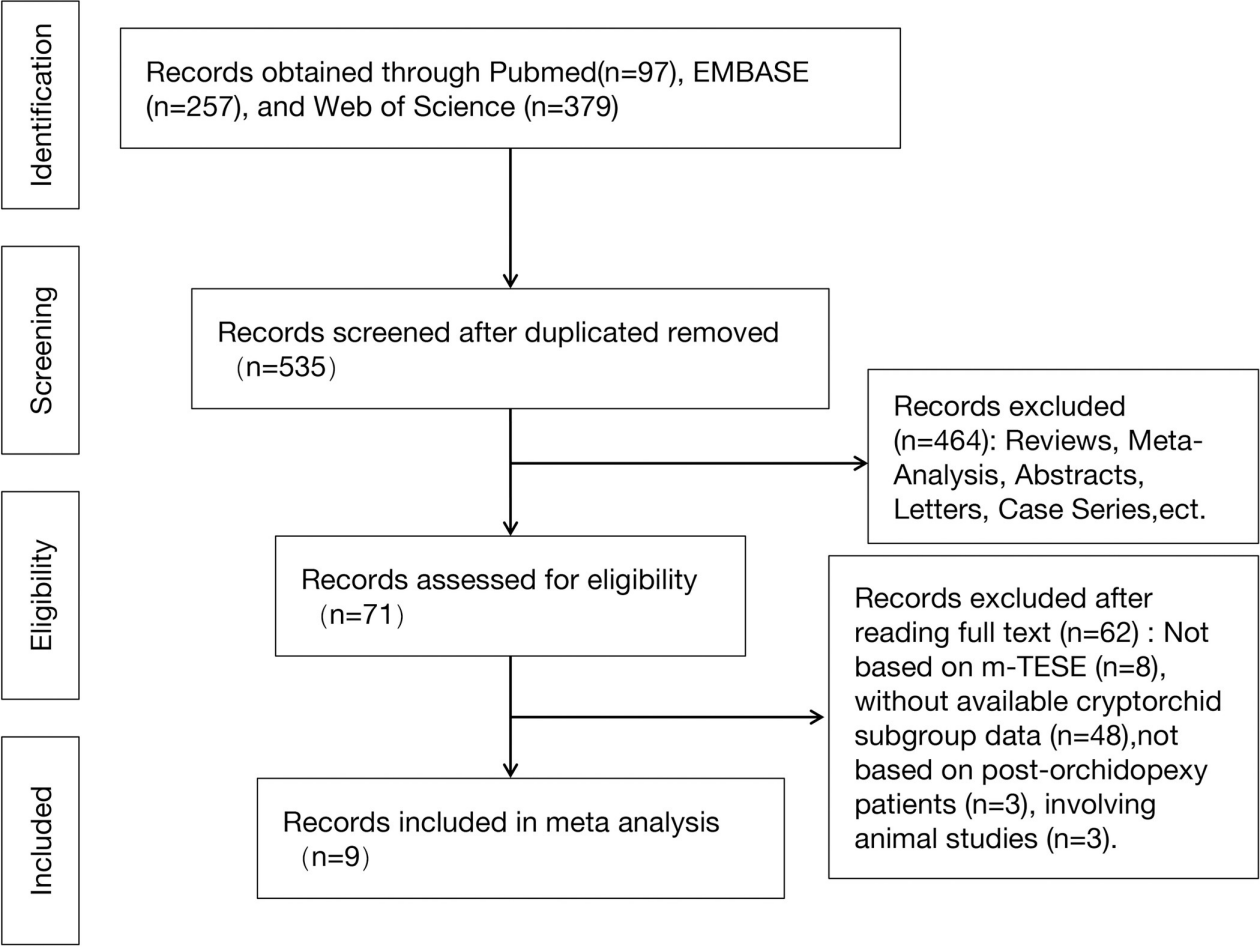
Flow diagram of the included studies in this meta-analysis.

Of the 538 articles identified, 9 studies were eligible and included in the meta-analysis [9–17]. Fig 1 (Fig 1) illustrates the identification and screening process for this study according to the PRISMA flowchart. A total of 935 patients were included in these 9 studies. Data from these studies were analyzed qualitatively and quantitatively (Table 1).

### Sperm retrieval rate

**Fig 2:**
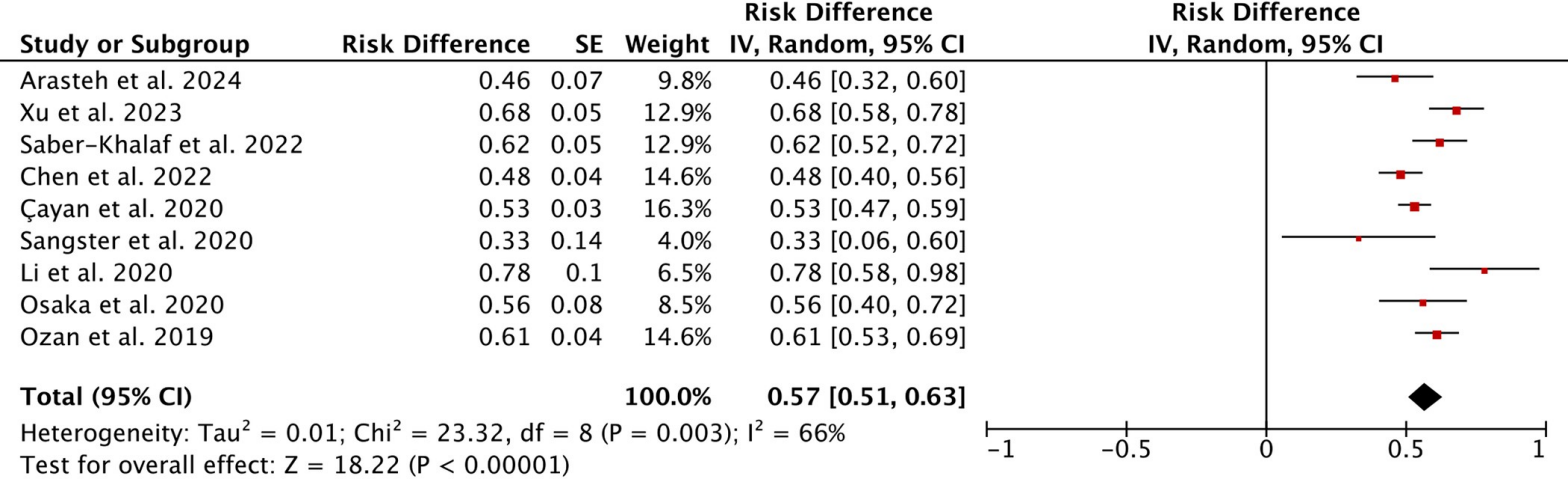
Forest plots of sperm retrieval rate under m-TESE. (a random-effects model was used).

Sperm Retrieval Rate (SRR) is the rate of patients with positive sperm retrieval outcomes in all patients undergoing sperm retrieval. A total of 9 studies comprising 935 patients who underwent m-TESE following surgery for azoospermia due to cryptorchidism were included. Egger’s test indicated no publication bias (P = 0.810). The overall SRR was approximately 57% (95% CI 51%–63%) (Fig 2). Significant heterogeneity was observed at 66%. Sensitivity analysis was conducted by excluding individual studies to evaluate the stability of the results.

### Age at orchidopexy/m-TESE

**Fig 3:**
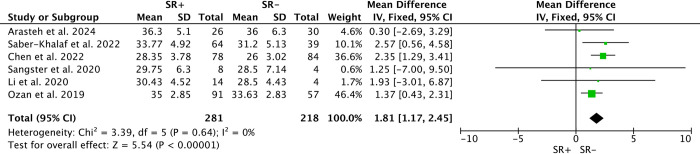
Forest plots of age at m-TESE between SR+ and SR-. Younger age at m-TESE was associated with lower sperm retrieval rates (SMD: 1.81; 95% CI 1.17–2.45; P<0.00001).

**Fig 4:**
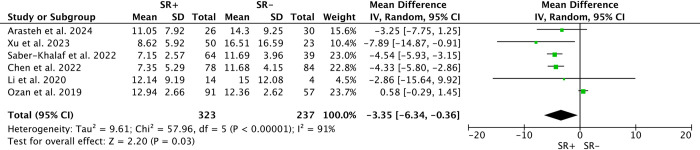
Forest plots of age at orchidopexy between SR+ and SR-. Younger age at orchidopexy was associated with higher sperm retrieval rates (WMD: -3.35; 95% CI -6.34 to -0.36; P<0.00001).

Six studies compared age differences in m-TESE outcomes between patients with successful sperm retrieval (SR+) and those without (SR-), totaling 281 SR+ and 218 SR-. Due to low heterogeneity (*I*^*2*^ = 0%), a fixed-effects model was employed. Younger age at m-TESE was associated with decreased likelihood of sperm retrieval (SMD: 1.81; 95% CI 1.17–2.45; P<0.00001) (Fig 3). In contrast, among 6 studies reporting age at orchidopexy, involving 323 SR+ and 237 SR-, younger age at orchidopexy was associated with higher success rates (WMD: -3.35; 95% CI -6.34 to -0.36; P<0.00001) (Fig 4), despite significant heterogeneity (*I*^*2*^ = 91%).

### Interval from orchidopexy to m-TESE

**Fig 5:**
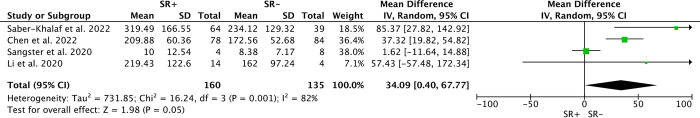
Forest plots of interval from orchidopexy to m-TESE between SR+ and SR-. Longer intervals from orchidopexy to m-TESE was associated with higher sperm retrieval rates(WMD: 34.09; 95% CI 0.40–67.77).(The unit of this data is months).

Four studies described the interval from orchidopexy to m-TESE and compared differences in interval duration between SR+ and SR- patients. The unit of this data is months. Due to high heterogeneity (*I*^*2*^ = 82%), a random-effects model was employed. SR+ patients tended to have longer intervals compared to SR- patients (WMD: 34.09; 95% CI 0.40–67.77) (Fig 5).

### Unilateral/Bilateral cryptorchidism

**Fig 6:**
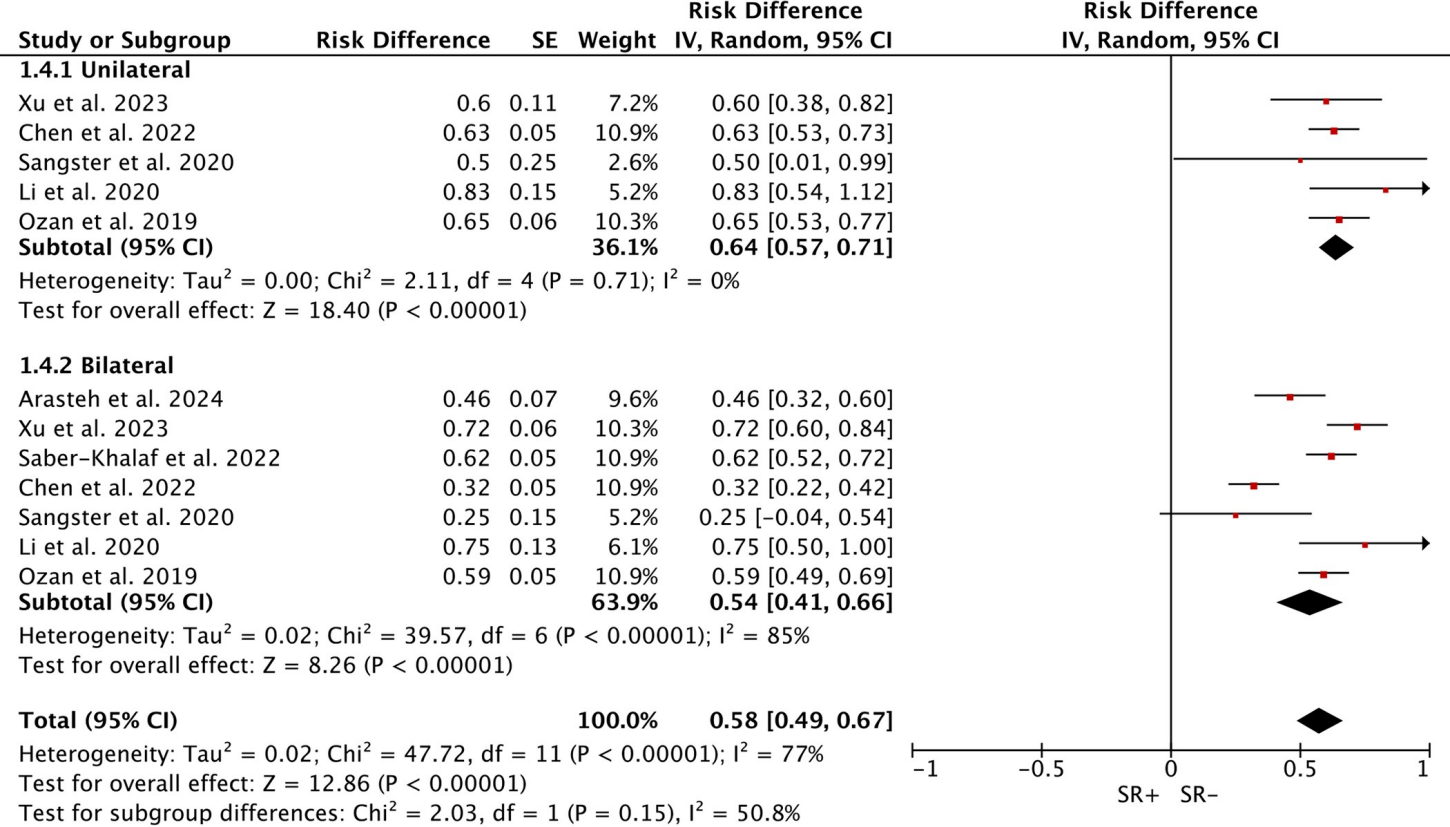
Forest plots of Unilateral/Bilateral between SR+ and SR-. No significant difference in sperm retrieval rates between unilateral and bilateral cases.

Five studies (178 cases) addressed SRR after unilateral cryptorchidism (113 SR+), and seven studies (394 cases) after bilateral cryptorchidism (214 SR+). Due to high heterogeneity (*I*^*2*^ = 77%), comparison using a random-effects model showed no significant difference in SRR between unilateral (WMD: 64%; 95% CI 57%–71%) and bilateral cases (WMD: 54%; 95% CI 41%–66%; P = 0.15) (Fig 6).

### Location of undescended testes

**Fig 7:**
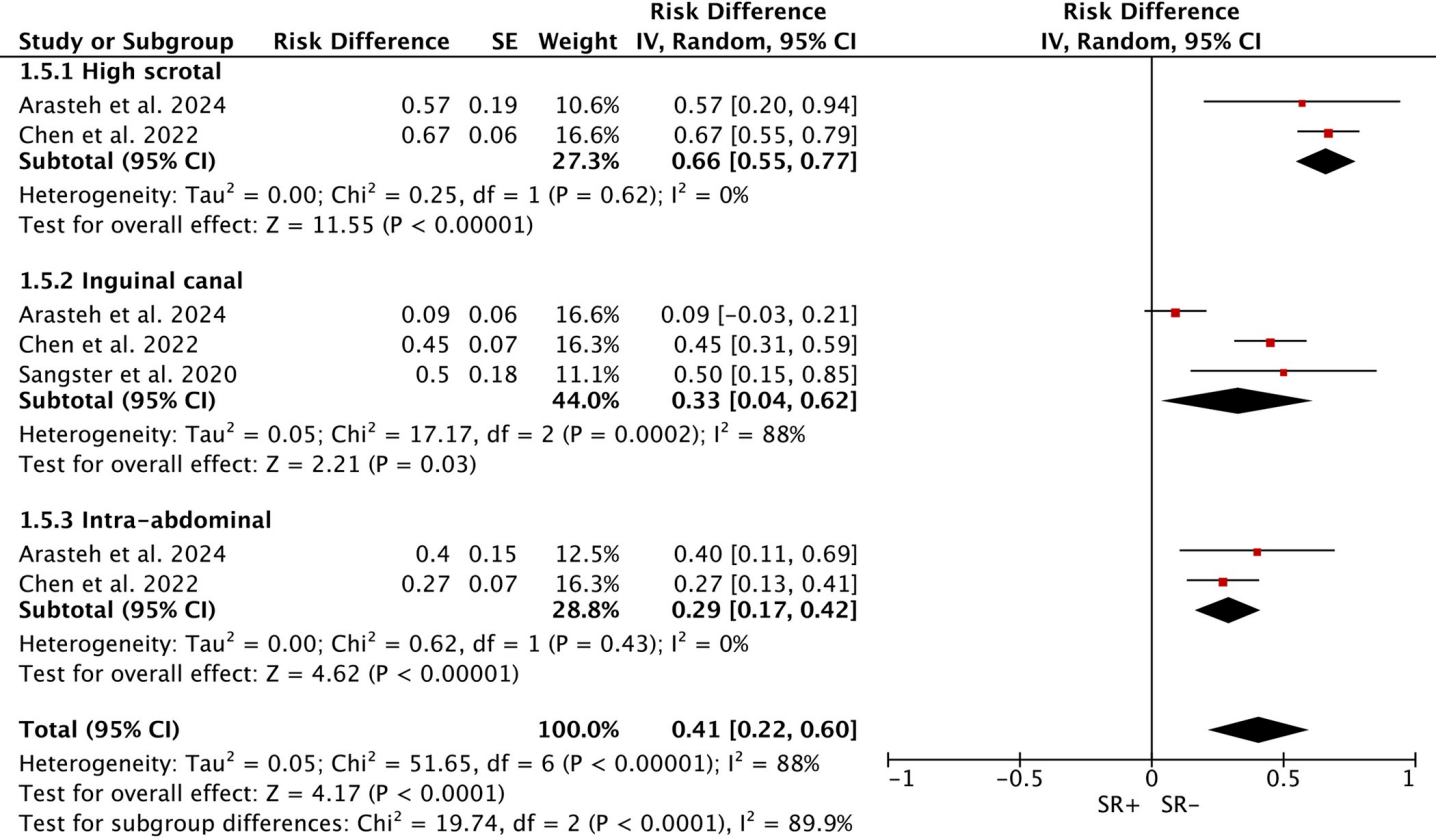
Forest plots of different locations of undescended testes between SR+ and SR-. High scrotal (66%) cryptorchidism was associated with higher sperm retrieval rates compared to inguinal canal (33%) and intra-abdominal (29%).

Three studies explored the relation between the location of cryptorchidism pre-orchidopexy and SRR, including high scrotal (67 cases), inguinal canal (89 cases), and intra-abdominal (54 cases). Due to high heterogeneity (*I*^*2*^ = 85.8%), a random-effects model was used. Significant subgroup differences were found, with a higher SRR in high scrotal (66%) compared to inguinal (33%) and intra-abdominal (29%) cryptorchidism (Fig 7).

### T, FSH, LH

**Fig 8:**
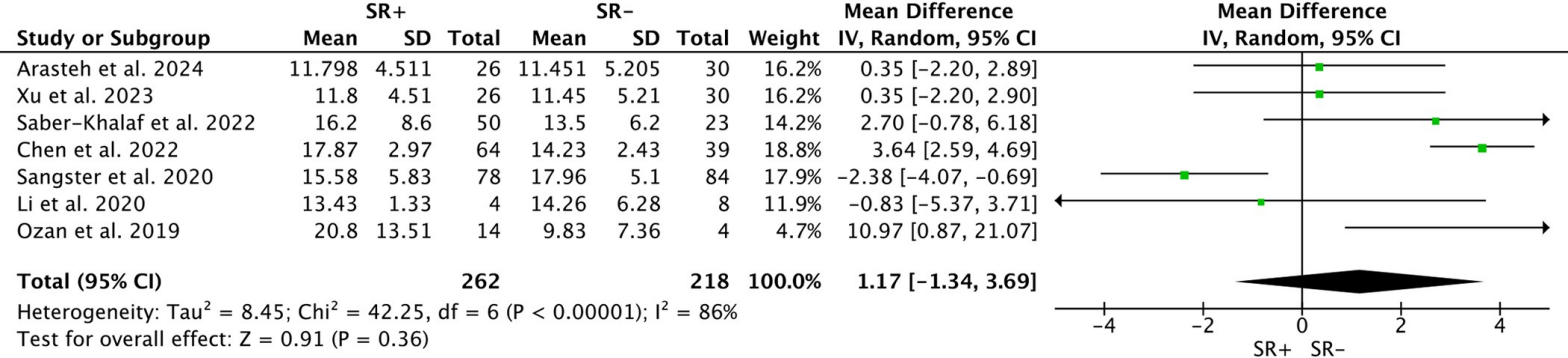
Forest plots of Testosterone between SR+ and SR-. No significant difference in testosterone levels between SR+ and SR- patients.

**Fig 9:**
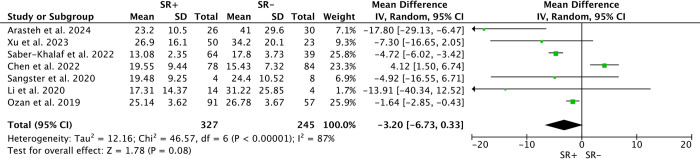
Forest plots of Follicle-Stimulating Hormone between SR+ and SR-. No significant difference in follicle-stimulating hormone levels between SR+ and SR- patients.

**Fig 10:**
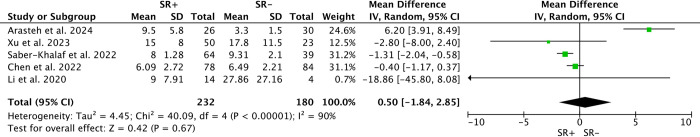
Forest plots of Luteinizing Hormone between SR+ and SR-. No significant difference in luteinizing hormone levels between SR+ and SR- patients.

Seven studies examined pre-m-TESE levels of Testosterone (T) and Follicle-Stimulating Hormone (FSH) among 327 SR+ and 245 SR-. Random-effects models found no significant differences in T levels (WMD: 1.17; 95% CI -1.34 to 3.69; P>0.05; *I*^*2*^ = 86%) or FSH levels (WMD: -3.20; 95% CI -6.73 to 0.33; P>0.05; *I*^*2*^ = 87%) between SR+ and SR- patients(Figs 8 and 9). Similarly, analysis of Luteinizing Hormone (LH) levels among 232 SR+ and 180 SR- patients across 5 studies showed no significant differences (WMD: 0.50; 95% CI -1.84 to 2.85; P>0.05; *I*^*2*^ = 90%) (Fig 10).

### Testicular volume

**Fig 11:**
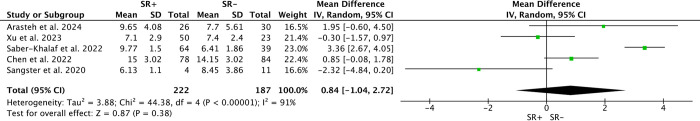
Forest plots of testicular volume between SR+ and SR-. No significant difference in testicular volume between SR+ and SR- patients.

Five studies compared testicular volume (TV) differences between 222 SR+ and 187 SR- patients. Due to high heterogeneity (*I*^*2*^ = 91%), a random-effects model was used, revealing no significant difference in TV between SR+ and SR- patients (WMD: 0.84; 95% CI -1.05 to 2.72; P>0.05) (Fig 11).

## Discussion

Cryptorchidism is among the most frequently occurring congenital conditions in male newborns, affecting approximately 1% to 4% of full-term infants, with an even higher incidence observed in preterm infants [1–3]. Higher temperatures in other areas compared to within the scrotum can negatively affect spermatogonia [18]. In children with cryptorchidism detected before the age of 1 year, human chorionic gonadotophin (HCG) and Luteinizing hormone-releasing hormone (LH-RH) may be tried, possibly inducing continued postnatal migration of the testicular gubernaculum, which leads to the descent of testis into the scrotum [19]. To optimize the chances of maintaining the production of sperm, it is typically advised that individuals with undescended testes undergo orchidopexy surgery no later than 12 months of age [5]. In patients with cryptorchidism diagnosed at puberty, the administration of human chorionic gonadotophin (HCG) or human menopausal gonadotropin (HMG) has been reported to enhance spermatogenesis in post-surgical patients [20].

However, even after pharmacological or surgical treatment, the incidence of azoospermia in patients with unilateral cryptorchidism ranges from 13–38%, and bilaterally up to 89% [21]. However, in this meta-analysis, there was no statistical difference in SRR between patients with unilateral cryptorchidism and those with bilateral cryptorchidism. This may be due to the fact that the patients included in this study had been diagnosed with azoospermia. The mechanisms by which cryptorchidism leads to spermatogenic dysfunction include impaired differentiation of gonocytes to Ad spermatogonia during micropuberty [22], impaired function of Sertoli and Leydig cells [23], and persistent hyperthermia causing oxidative stress and disruption of the blood-testis barrier [24,25], which ultimately leads to a reduction in the number of germ cells, or even azoospermia [26,27].

For azoospermia patients, especially those with non-obstructive azoospermia, it is almost impossible to impregnate the female partner through natural ways. In 1995, the first NOA patient underwent TESE and intracytoplasmic sperm injection (ICSI) and successfully impregnated the female partner, signalling a possible chance of fatherhood for patients with NOA [28]. However, conventional TESE obtains limited sperm in a single sampling, and multiple sampling removes a large amount of testicular tissue and damage to the testicular vasculature [29]. In order to minimise damage to the testicular vasculature and postoperative testicular hypoplasia, m-TESE was invented in 1998 by Schlegel et al.The advantage is under the microscope, the surgeon can identify differences in the seminiferous tubules and locate the ones potentially most probable to have sperm [30]. Now m-TESE is considered the gold standard procedure for sperm extraction in NOA patients.

But the sperm retrieval rate of m-TESE is uncertain and is affected by many factors. Etiology, preoperative serum sex hormone concentrations, testicular histopathology and some potential biomarkers have previously been considered as possible predictors of sperm retrieval rate, but few studies have specifically examined sperm retrieval rate in patients with cryptorchidism. We used meta-analysis to summarise the sperm retrieval rate in patients with cryptorchidism and tried to find out the factors that may affect the sperm retrieval rate in patients with cryptorchidism by subgroup analysis. This study has potential guidance for the management of patients with cryptorchidism.

Our systematic review and meta-analysis showed that the sperm retrieval rate in patients underwent m-TESE in post-orchidopexy patients was approximately 57% (95% CI 51%–63%). Patients with earlier orchidopexy, older age at m-TESE, larger intervals between m-TESE and orchidopexy, and undescended testes closer to the scrotum had higher sperm retrieval rates by m-TESE. There were no significant differences in unilateral/bilateral cryptorchidism, testicular volume, serum FSH, LH and T concentrations between SR+ and SR- patients.

Our meta-analysis showed that SR+ patients underwent orchidopexy at a younger age compared to SR- patients (WMD: -3.35; 95% CI -6.34–0.36), suggesting that earlier administration of orchidopexy could increase the success rate of sperm retrieval in m-TESE. Berkowitz et al. showed that spontaneous descent of cryptorchid testes is almost impossible after 6 months [31]. Wilkerson and Cortes, Kollin et al. showed that from 9 months onwards, the undescended testes decrease in size compared to the normal testes and lose a large number of germ cells [32–34]. A meta-analysis by Allin et al. included 15 studies of orchidopexy performed before/after 1 year of age and showed that surgery performed before 1 year of age had a better fertility potential and did not increase the risk of testicular atrophy [35]. The EAU guidelines recommends orchidopexy before 18 months to avoid loss of potential fertility [5]. Our meta-analysis focused on patients with cryptorchidism combined with azoospermia. The mean age of these patients underwent orchidopexy was over 18 months, but these patients still have the potential for successful sperm retrieval through m-TESE. Earlier orchidopexy improves the success rate of sperm retrieval, so it seems that early orchidopexy should still be recommended for patients with cryptorchidism older than 18 months.

After orchidopexy, our meta-analysis showed that longer intervals, as well as later performance of m-TESE were associated with higher sperm retrieval rates in m-TESE. This may be explained by the correction of the spermatogenic environment after undergoing orchidopexy, which may help the residual seminiferous tubules to produce spermatozoa. Nevertheless, in patients with cryptorchidism and azoospermia, there is not enough evidence to recommend how soon should m-TESE be performed after orchidopexy.

Our meta-analysis showed that the closer the undescended testis was to the scrotum before orchidopexy, the higher the sperm retrieval rate, and the closer to the abdominal cavity, the lower the sperm retrieval rate. Xu et al. showed that testosterone after HCG stimulation, AMH, inhibin B was significantly lower in patients with high undescended testis than with the low undescended testis group, suggesting that Leydig and Sertoli cell function are both more significantly damaged in patients with high undescended testis [36]. The reason for this is probably that the closer to the abdominal cavity, the higher temperature the testicles are exposed. Testis is a temperature-sensitive organ, and prolonged high testicular temperatures induce oxidative stress [24], disruption of Sertoli intercellular tight junctions and blood-testis barrier [25], which triggers germ cell apoptosis, reduces testicular size, and leads to spermatogonial reduction [26,27]. Another explanation related to surgery is that the higher undescended testis, the more collateral vessels need to be dissected and higher tension during surgery, and greater probability of postoperative testicular ischaemia and testicular atrophy [37]. Thus, higher undescended testis have lower sperm retrieval rates post-orchidopexy. The necessity of orchidopexy for high, especially intra-abdominal testes is still controversial.

Our meta-analysis showed no difference between unilateral or bilateral cryptorchidism on the outcome of m-TESE. The prevalence of combined azoospermia in patients with unilateral cryptorchidism ranges from 13–38% [21], implying that the contralateral testis, which has naturally descended, is also damaged, which may be due to a failure in the differentiation of Ad spermatogonia on both sides during mini-puberty [22]. During this period, male reproductive potential is developed within the first 30–90 days of life, gonocytes differentiate into Ad spermatogonia, which establish male germ cell memory and male-specific DNA methylation pathways [38]. Hadziselimovic et al. analysed 31 patients underwent orchidopexy and testicular biopsy through 20 years of follow-up, revealing that the lack of Ad spermatogonia in infancy is highly correlated with spermatogenic disorders in adulthood, suggesting that the differentiation of gonocytes into Ad spermatogonia seems to be the best predictor of fertility potential, while in patients with cryptorchidism the differentiation process may be impaired [39]. The process requires the involvement of gonadotropins and testosterone [40]. However, the results of a study by Gendrel et al. of 154 boys with cryptorchidism aged 1 month ~ 15 years showed that serum LH concentrations and LH concentrations in response to LH-RH were significantly lower in boys with cryptorchidism than in controls [41]. This is associated with Leydig cell atrophy and failure of gonocytes differentiate into Ad spermatogonia in the testis [22]. In patients with unilateral cryptorchidism, in addition to local anatomical factors, there may be gonadotropin insufficiency or insufficient response to gonadotropins, so the contralateral "normal" testicle may also have spermatogenic disorders in patients with unilateral cryptorchidism [42,43].

Furthermore, our meta-analysis did not find differences in testicular volume, serum FSH, LH and T concentrations between SR+ and SR- patients. Of the five articles mentioning testicular volume, only the study by Saber-Khalaf et al. found that SR+ patients had greater testicular volume [11], while the remaining four articles did not find any statistical difference in testicular volume. And serum FSH, LH and testosterone concentrations all had conflicting findings between SR+ and SR- patients. Although higher gonadotropin concentrations and lower testicular volume and testosterone may predict further testicular hypoplasia, there appears to be no significant correlation with sperm retrieval outcomes. Hormone levels are only representative of the general condition of the testes and do not imply that no seminiferous tubules are capable of producing sperm.

Our study aimed to compare the differences in clinical parameters in post-orchidopexy patients with different m-TESE outcomes by meta-analysis. However, there are inevitably some limitations. First, only nine studies were included and all were retrospective. In addition, these studies mainly included Asian and European countries, with regions such as the Americas and Africa missing. Although no publication bias was detected by Egger’s test, the geographical factor makes the results of the studies lack generalisability. Next, the studies did not provide a detailed description of pharmacological treatments before and after orchidopexy, so the impact of pharmacological interventions could not be analysed. Finally, the heterogeneity of the studies was high, and although we looked for sources of heterogeneity as much as possible through subgroup analyses and concluded to some extent, there may still be some differences that cannot be accurately expressed.

In patients with undetected cryptorchidism and azoospermia after puberty, better pharmacological treatments and optimal interval from orchidopexy to m-TESE still requires further studies. More researchs are still needed on why patients with unilateral cryptorchidism suffer from azoospermia. The mechanism of how unilateral undescended testis affects the contralateral normal testis needs to be explored.

Overall, with this meta-analysis, we can be informed that successful sperm retrieval outcomes are associated with earlier orchidopexy and later m-TESE in azoospermic patients post-orchidopexy. Therefore, intervention for cryptorchidism should be done as early as possible, whether it is performed before 18 months of age or detected at a much older age; it should be taken seriously and done as early as possible.

## Conclusions

To maximize fertility potential in patients with cryptorchidism, orchidopexy should be performed as early as possible, ideally before 18 months of age, but if not feasible, it should be done before the age of 10. In patients with undetected cryptorchidism and azoospermia after puberty, m-TESE should not be performed immediately after orchidopexy, optimal interval from orchidopexy to m-TESE still requires further study. Undescended testis closer to the scrotum, increase the sperm retrieval rate of m-TESE in post-orchidopexy cryptorchid patients.

## Supporting information

S1 File The PRISMA 2020 statement. (DOCX)

S2 File Descriptions of 535 excluded records and reasons. (XLS)

S3 File Descriptions of studies included in the meta-analysis. (XLS)

S4 File Quality assessments for each study in the meta-analysis. (DOC)

S5 File Search terms. (DOC)

S6 File Funnel plots for each parameter. (DOC)
