# Supplementary material for: Microdissection testicular sperm extraction outcomes in azoospermic patients post-orchidopexy surgery: A systematic review and meta-analysis
Source: PLoS One. 2024 Nov 15;19(11):e0313866. doi: 10.1371/journal.pone.0313866 (PMC11567534; doi:10.1371/journal.pone.0313866)
Supplement: S4 File — (DOC) [file pone.0313866.s004.doc]

**Quality Assessment Tool for Before-After (Pre-Post) Studies With No Control Group**

Study: Cayan S, Orhan I, Altay B, Asci R, Akbay E, Ayas B, et al. Fertility outcomes and predictors for successful sperm retrieval and pregnancy in 327 azoospermic men with a history of cryptorchidism who underwent microdissection testicular sperm extraction. Andrology. 2021;9(1):253-9. Epub 20201008. doi: 10.1111/andr.12910. PubMed PMID: 32960506.

| Criteria | Yes | No | Other (CD,NR, NA)* | Additional Comments |
| --- | --- | --- | --- | --- |
| 1. Was the study question or objective clearly stated? | √ |  |  |  |
| 2. Were eligibility/selection criteria for the study population prespecified and clearly described? | √ |  |  |  |
| 3. Were the participants in the study representative of those who would be eligible for the test/service/intervention in the general or clinical population of interest? | √ |  |  |  |
| 4. Were all eligible participants that met the prespecified entry criteria enrolled? | √ |  |  |  |
| 5. Was the sample size sufficiently large to provide confidence in the findings? |  | √ |  |  |
| 6. Was the test/service/intervention clearly described and delivered consistently across the study population? | √ |  |  |  |
| 7. Were the outcome measures prespecified, clearly defined, valid, reliable, and assessed consistently across all study participants? | √ |  |  |  |
| 8. Were the people assessing the outcomes blinded to the participants' exposures/interventions? |  | √ |  |  |
| 9. Was the loss to follow-up after baseline 20% or less? Were those lost to follow-up accounted for in the analysis? | √ |  |  |  |
| 10. Did the statistical methods examine changes in outcome measures from before to after the intervention? Were statistical tests done that provided p values for the pre-to-post changes? | √ |  |  |  |
| 11. Were outcome measures of interest taken multiple times before the intervention and multiple times after the intervention (i.e., did they use an interrupted time-series design)? | √ |  |  |  |
| 12. If the intervention was conducted at a group level (e.g., a whole hospital, a community, etc.) did the statistical analysis take into account the use of individual-level data to determine effects at the group level? |  |  | NA | No group-level interventions. |

Quality Rating (Good, Fair, or Poor)

Rater #1 (Haonan He) Initials:Good

Rater #2 (Hong Xiao) Initials:Good

*CD, cannot determine; NA, not applicable; NR, not reported

**Quality Assessment Tool for Before-After (Pre-Post) Studies With No Control Group**

Study: Sangster P, Alnajjar HM, Ahmed K, Christodoulidou M, Williamson E, Kelly JD, et al. Microdissection TESE (mTESE) following adult orchidopexy for undescended intra-abdominal and inguinal testicles - surgical techniques and outcomes from a single-centre cohort. Andrology. 2020;8(1):166-70. Epub 20190710. doi: 10.1111/andr.12679. PubMed PMID: 31293079.

| Criteria | Yes | No | Other (CD,NR, NA)* | Additional Comments |
| --- | --- | --- | --- | --- |
| 1. Was the study question or objective clearly stated? | √ |  |  |  |
| 2. Were eligibility/selection criteria for the study population prespecified and clearly described? | √ |  |  |  |
| 3. Were the participants in the study representative of those who would be eligible for the test/service/intervention in the general or clinical population of interest? |  | √ |  |  |
| 4. Were all eligible participants that met the prespecified entry criteria enrolled? | √ |  |  |  |
| 5. Was the sample size sufficiently large to provide confidence in the findings? |  | √ |  |  |
| 6. Was the test/service/intervention clearly described and delivered consistently across the study population? | √ |  |  |  |
| 7. Were the outcome measures prespecified, clearly defined, valid, reliable, and assessed consistently across all study participants? | √ |  |  |  |
| 8. Were the people assessing the outcomes blinded to the participants' exposures/interventions? |  | √ |  |  |
| 9. Was the loss to follow-up after baseline 20% or less? Were those lost to follow-up accounted for in the analysis? | √ |  |  |  |
| 10. Did the statistical methods examine changes in outcome measures from before to after the intervention? Were statistical tests done that provided p values for the pre-to-post changes? | √ |  |  |  |
| 11. Were outcome measures of interest taken multiple times before the intervention and multiple times after the intervention (i.e., did they use an interrupted time-series design)? |  | √ |  |  |
| 12. If the intervention was conducted at a group level (e.g., a whole hospital, a community, etc.) did the statistical analysis take into account the use of individual-level data to determine effects at the group level? |  |  | NA | No group-level interventions. |

Quality Rating (Good, Fair, or Poor)

Rater #1 (Haonan He) Initials:Good

Rater #2 (Hong Xiao) Initials:Fair

*CD, cannot determine; NA, not applicable; NR, not reported

**Quality Assessment Tool for Before-After (Pre-Post) Studies With No Control Group**

Study: Saber-Khalaf M, Ali AF, Elsoghier OM. Predictive factors of successful testicular sperm extraction for non-obstructive azoospermia with a history of bilateral cryptorchidism and normal testosterone. Andrologia. 2022;54(1):e14284. Epub 20211018. doi: 10.1111/and.14284. PubMed PMID: 34664297.

| Criteria | Yes | No | Other (CD,NR, NA)* | Additional Comments |
| --- | --- | --- | --- | --- |
| 1. Was the study question or objective clearly stated? | √ |  |  |  |
| 2. Were eligibility/selection criteria for the study population prespecified and clearly described? | √ |  |  |  |
| 3. Were the participants in the study representative of those who would be eligible for the test/service/intervention in the general or clinical population of interest? | √ |  |  |  |
| 4. Were all eligible participants that met the prespecified entry criteria enrolled? | √ |  |  |  |
| 5. Was the sample size sufficiently large to provide confidence in the findings? |  | √ |  |  |
| 6. Was the test/service/intervention clearly described and delivered consistently across the study population? | √ |  |  |  |
| 7. Were the outcome measures prespecified, clearly defined, valid, reliable, and assessed consistently across all study participants? | √ |  |  |  |
| 8. Were the people assessing the outcomes blinded to the participants' exposures/interventions? |  | √ |  |  |
| 9. Was the loss to follow-up after baseline 20% or less? Were those lost to follow-up accounted for in the analysis? | √ |  |  |  |
| 10. Did the statistical methods examine changes in outcome measures from before to after the intervention? Were statistical tests done that provided p values for the pre-to-post changes? | √ |  |  |  |
| 11. Were outcome measures of interest taken multiple times before the intervention and multiple times after the intervention (i.e., did they use an interrupted time-series design)? | √ |  |  |  |
| 12. If the intervention was conducted at a group level (e.g., a whole hospital, a community, etc.) did the statistical analysis take into account the use of individual-level data to determine effects at the group level? |  |  | NA | No group-level interventions. |

Quality Rating (Good, Fair, or Poor)

Rater #1 (Haonan He) Initials:Good

Rater #2 (Hong Xiao) Initials:Good

*CD, cannot determine; NA, not applicable; NR, not reported

**Quality Assessment Tool for Before-After (Pre-Post) Studies With No Control Group**

Study: Li P, Yao CC, Zhi EL, Xu Y, Wan Z, Jiang YC, et al. Modified stepwise mini-incision microdissection testicular sperm extraction: a useful technique for patients with a history of orchidopexy affected by non-obstructive azoospermia. J Zhejiang Univ Sci B. 2020;21(1):87-92. doi: 10.1631/jzus.B1900232. PubMed PMID: 31898445; PubMed Central PMCID: PMCPMC6964994.

| Criteria | Yes | No | Other (CD,NR, NA)* | Additional Comments |
| --- | --- | --- | --- | --- |
| 1. Was the study question or objective clearly stated? | √ |  |  |  |
| 2. Were eligibility/selection criteria for the study population prespecified and clearly described? | √ |  |  |  |
| 3. Were the participants in the study representative of those who would be eligible for the test/service/intervention in the general or clinical population of interest? | √ |  |  |  |
| 4. Were all eligible participants that met the prespecified entry criteria enrolled? | √ |  |  |  |
| 5. Was the sample size sufficiently large to provide confidence in the findings? |  | √ |  |  |
| 6. Was the test/service/intervention clearly described and delivered consistently across the study population? | √ |  |  |  |
| 7. Were the outcome measures prespecified, clearly defined, valid, reliable, and assessed consistently across all study participants? | √ |  |  |  |
| 8. Were the people assessing the outcomes blinded to the participants' exposures/interventions? |  | √ |  |  |
| 9. Was the loss to follow-up after baseline 20% or less? Were those lost to follow-up accounted for in the analysis? | √ |  |  |  |
| 10. Did the statistical methods examine changes in outcome measures from before to after the intervention? Were statistical tests done that provided p values for the pre-to-post changes? | √ |  |  |  |
| 11. Were outcome measures of interest taken multiple times before the intervention and multiple times after the intervention (i.e., did they use an interrupted time-series design)? | √ |  |  |  |
| 12. If the intervention was conducted at a group level (e.g., a whole hospital, a community, etc.) did the statistical analysis take into account the use of individual-level data to determine effects at the group level? |  |  | NA | No group-level interventions. |

Quality Rating (Good, Fair, or Poor)

Rater #1 (Haonan He) Initials:Good

Rater #2 (Hong Xiao) Initials:Good

*CD, cannot determine; NA, not applicable; NR, not reported

**Quality Assessment Tool for Before-After (Pre-Post) Studies With No Control Group**

Study: Ozan T, Karakeci A, Kaplancan T, Pirincci N, Firdolas F, Orhan I. Are predictive factors in sperm retrieval and pregnancy rates present in nonobstructive azoospermia patients by microdissection testicular sperm extraction on testicle with a history of orchidopexy operation? Andrologia. 2019;51(11):e13430. Epub 20191001. doi: 10.1111/and.13430. PubMed PMID: 31573111.

| Criteria | Yes | No | Other (CD,NR, NA)* | Additional Comments |
| --- | --- | --- | --- | --- |
| 1. Was the study question or objective clearly stated? | √ |  |  |  |
| 2. Were eligibility/selection criteria for the study population prespecified and clearly described? | √ |  |  |  |
| 3. Were the participants in the study representative of those who would be eligible for the test/service/intervention in the general or clinical population of interest? | √ |  |  |  |
| 4. Were all eligible participants that met the prespecified entry criteria enrolled? | √ |  |  |  |
| 5. Was the sample size sufficiently large to provide confidence in the findings? |  | √ |  |  |
| 6. Was the test/service/intervention clearly described and delivered consistently across the study population? | √ |  |  |  |
| 7. Were the outcome measures prespecified, clearly defined, valid, reliable, and assessed consistently across all study participants? | √ |  |  |  |
| 8. Were the people assessing the outcomes blinded to the participants' exposures/interventions? |  | √ |  |  |
| 9. Was the loss to follow-up after baseline 20% or less? Were those lost to follow-up accounted for in the analysis? | √ |  |  |  |
| 10. Did the statistical methods examine changes in outcome measures from before to after the intervention? Were statistical tests done that provided p values for the pre-to-post changes? | √ |  |  |  |
| 11. Were outcome measures of interest taken multiple times before the intervention and multiple times after the intervention (i.e., did they use an interrupted time-series design)? | √ |  |  |  |
| 12. If the intervention was conducted at a group level (e.g., a whole hospital, a community, etc.) did the statistical analysis take into account the use of individual-level data to determine effects at the group level? |  |  | NA | No group-level interventions. |

Quality Rating (Good, Fair, or Poor)

Rater #1 (Haonan He) Initials:Good

Rater #2 (Hong Xiao) Initials:Good

*CD, cannot determine; NA, not applicable; NR, not reported

**Quality Assessment Tool for Before-After (Pre-Post) Studies With No Control Group**

Study: Arasteh H, Gilani MAS, Ramezani-Binabaj M, Babaei M. Microdissection testicular sperm extraction outcomes in azoospermic patients with bilateral orchidopexy. Andrology. 2024;12(1):157-63. Epub 20230602. doi: 10.1111/andr.13463. PubMed PMID: 37210678.

| Criteria | Yes | No | Other (CD,NR, NA)* | Additional Comments |
| --- | --- | --- | --- | --- |
| 1. Was the study question or objective clearly stated? | √ |  |  |  |
| 2. Were eligibility/selection criteria for the study population prespecified and clearly described? | √ |  |  |  |
| 3. Were the participants in the study representative of those who would be eligible for the test/service/intervention in the general or clinical population of interest? |  | √ |  |  |
| 4. Were all eligible participants that met the prespecified entry criteria enrolled? | √ |  |  |  |
| 5. Was the sample size sufficiently large to provide confidence in the findings? |  | √ |  |  |
| 6. Was the test/service/intervention clearly described and delivered consistently across the study population? | √ |  |  |  |
| 7. Were the outcome measures prespecified, clearly defined, valid, reliable, and assessed consistently across all study participants? | √ |  |  |  |
| 8. Were the people assessing the outcomes blinded to the participants' exposures/interventions? |  | √ |  |  |
| 9. Was the loss to follow-up after baseline 20% or less? Were those lost to follow-up accounted for in the analysis? | √ |  |  |  |
| 10. Did the statistical methods examine changes in outcome measures from before to after the intervention? Were statistical tests done that provided p values for the pre-to-post changes? | √ |  |  |  |
| 11. Were outcome measures of interest taken multiple times before the intervention and multiple times after the intervention (i.e., did they use an interrupted time-series design)? | √ |  |  |  |
| 12. If the intervention was conducted at a group level (e.g., a whole hospital, a community, etc.) did the statistical analysis take into account the use of individual-level data to determine effects at the group level? |  |  | NA | No group-level interventions. |

Quality Rating (Good, Fair, or Poor)

Rater #1 (Haonan He) Initials:Good

Rater #2 (Hong Xiao) Initials:Good

*CD, cannot determine; NA, not applicable; NR, not reported

**Quality Assessment Tool for Before-After (Pre-Post) Studies With No Control Group**

Study: Chen XL, Wei YA, Ren XH, Zhang X, Li GY, Lu ZW, et al. Predictive factors for successful sperm retrieval by microdissection testicular sperm extraction in men with nonobstructive azoospermia and a history of cryptorchidism. Asian J Androl. 2022;24(5):503-8. doi: 10.4103/aja2021102. PubMed PMID: 35042308; PubMed Central PMCID: PMCPMC9491033.

| Criteria | Yes | No | Other (CD,NR, NA)* | Additional Comments |
| --- | --- | --- | --- | --- |
| 1. Was the study question or objective clearly stated? | √ |  |  |  |
| 2. Were eligibility/selection criteria for the study population prespecified and clearly described? | √ |  |  |  |
| 3. Were the participants in the study representative of those who would be eligible for the test/service/intervention in the general or clinical population of interest? | √ |  |  |  |
| 4. Were all eligible participants that met the prespecified entry criteria enrolled? | √ |  |  |  |
| 5. Was the sample size sufficiently large to provide confidence in the findings? |  | √ |  |  |
| 6. Was the test/service/intervention clearly described and delivered consistently across the study population? | √ |  |  |  |
| 7. Were the outcome measures prespecified, clearly defined, valid, reliable, and assessed consistently across all study participants? | √ |  |  |  |
| 8. Were the people assessing the outcomes blinded to the participants' exposures/interventions? |  | √ |  |  |
| 9. Was the loss to follow-up after baseline 20% or less? Were those lost to follow-up accounted for in the analysis? | √ |  |  |  |
| 10. Did the statistical methods examine changes in outcome measures from before to after the intervention? Were statistical tests done that provided p values for the pre-to-post changes? | √ |  |  |  |
| 11. Were outcome measures of interest taken multiple times before the intervention and multiple times after the intervention (i.e., did they use an interrupted time-series design)? | √ |  |  |  |
| 12. If the intervention was conducted at a group level (e.g., a whole hospital, a community, etc.) did the statistical analysis take into account the use of individual-level data to determine effects at the group level? |  |  | NA | No group-level interventions. |

Quality Rating (Good, Fair, or Poor)

Rater #1 (Haonan He) Initials:Good

Rater #2 (Hong Xiao) Initials:Good

*CD, cannot determine; NA, not applicable; NR, not reported

**Quality Assessment Tool for Before-After (Pre-Post) Studies With No Control Group**

Study: Xu S, Huang Y, Yao C, Li P, Zhi E, Chen W, et al. Stepwise mini-incision microdissection testicular sperm extraction in NOA patients with a history of cryptorchidism: a case-control study. Basic Clin Androl. 2023;33(1):21. Epub 20230817. doi: 10.1186/s12610-023-00196-w. PubMed PMID: 37587426; PubMed Central PMCID: PMCPMC10433673.

| Criteria | Yes | No | Other (CD,NR, NA)* | Additional Comments |
| --- | --- | --- | --- | --- |
| 1. Was the study question or objective clearly stated? | √ |  |  |  |
| 2. Were eligibility/selection criteria for the study population prespecified and clearly described? | √ |  |  |  |
| 3. Were the participants in the study representative of those who would be eligible for the test/service/intervention in the general or clinical population of interest? |  | √ |  |  |
| 4. Were all eligible participants that met the prespecified entry criteria enrolled? | √ |  |  |  |
| 5. Was the sample size sufficiently large to provide confidence in the findings? |  | √ |  |  |
| 6. Was the test/service/intervention clearly described and delivered consistently across the study population? | √ |  |  |  |
| 7. Were the outcome measures prespecified, clearly defined, valid, reliable, and assessed consistently across all study participants? | √ |  |  |  |
| 8. Were the people assessing the outcomes blinded to the participants' exposures/interventions? |  | √ |  |  |
| 9. Was the loss to follow-up after baseline 20% or less? Were those lost to follow-up accounted for in the analysis? | √ |  |  |  |
| 10. Did the statistical methods examine changes in outcome measures from before to after the intervention? Were statistical tests done that provided p values for the pre-to-post changes? | √ |  |  |  |
| 11. Were outcome measures of interest taken multiple times before the intervention and multiple times after the intervention (i.e., did they use an interrupted time-series design)? | √ |  |  |  |
| 12. If the intervention was conducted at a group level (e.g., a whole hospital, a community, etc.) did the statistical analysis take into account the use of individual-level data to determine effects at the group level? |  |  | NA | No group-level interventions. |

Quality Rating (Good, Fair, or Poor)

Rater #1 (Haonan He) Initials:Good

Rater #2 (Hong Xiao) Initials:Good

*CD, cannot determine; NA, not applicable; NR, not reported

**Quality Assessment Tool for Before-After (Pre-Post) Studies With No Control Group**

Study: Osaka A, Iwahata T, Kobori Y, Shimomura Y, Yoshikawa N, Onota S, et al. Testicular volume in non-obstructive azoospermia with a history of bilateral cryptorchidism may predict successful sperm retrieval by testicular sperm extraction. Reprod Med Biol. 2020;19(4):372-7. Epub 20200712. doi: 10.1002/rmb2.12338. PubMed PMID: 33071639; PubMed Central PMCID: PMCPMC7542013.

| Criteria | Yes | No | Other (CD,NR, NA)* | Additional Comments |
| --- | --- | --- | --- | --- |
| 1. Was the study question or objective clearly stated? | √ |  |  |  |
| 2. Were eligibility/selection criteria for the study population prespecified and clearly described? | √ |  |  |  |
| 3. Were the participants in the study representative of those who would be eligible for the test/service/intervention in the general or clinical population of interest? |  | √ |  |  |
| 4. Were all eligible participants that met the prespecified entry criteria enrolled? | √ |  |  |  |
| 5. Was the sample size sufficiently large to provide confidence in the findings? |  | √ |  |  |
| 6. Was the test/service/intervention clearly described and delivered consistently across the study population? | √ |  |  |  |
| 7. Were the outcome measures prespecified, clearly defined, valid, reliable, and assessed consistently across all study participants? | √ |  |  |  |
| 8. Were the people assessing the outcomes blinded to the participants' exposures/interventions? |  | √ |  |  |
| 9. Was the loss to follow-up after baseline 20% or less? Were those lost to follow-up accounted for in the analysis? | √ |  |  |  |
| 10. Did the statistical methods examine changes in outcome measures from before to after the intervention? Were statistical tests done that provided p values for the pre-to-post changes? | √ |  |  |  |
| 11. Were outcome measures of interest taken multiple times before the intervention and multiple times after the intervention (i.e., did they use an interrupted time-series design)? |  | √ |  |  |
| 12. If the intervention was conducted at a group level (e.g., a whole hospital, a community, etc.) did the statistical analysis take into account the use of individual-level data to determine effects at the group level? |  |  | NA | No group-level interventions. |

Quality Rating (Good, Fair, or Poor)

Rater #1 (Haonan He) Initials:Fair

Rater #2 (Hong Xiao) Initials:Good

*CD, cannot determine; NA, not applicable; NR, not reported
