# Supplementary material for: Microdissection testicular sperm extraction outcomes in azoospermic patients post-orchidopexy surgery: A systematic review and meta-analysis
Source: PLoS One. 2024 Nov 15;19(11):e0313866. doi: 10.1371/journal.pone.0313866 (PMC11567534; doi:10.1371/journal.pone.0313866)
Supplement: S5 File — (DOC) [file pone.0313866.s005.doc]

**EMBASE**

(‘sperm retrieval rates’ OR ‘testicular sperm extraction’ OR ’micro dissection testicular sperm extraction’ OR ‘Micro-TESE’ OR ’M-TESE’) AND (‘Cryptorchidism’ OR ‘Cryptorchism’ OR ‘Testes, Undescended)’ OR ‘Undescended Testes’ OR 'Testis, Undescended' OR 'cryptorchid' OR 'Cryptorchidism, Unilateral Or Bilateral' OR 'Undescended Testis' OR 'Bilateral Cryptorchidism' OR 'Cryptorchidism, Bilateral' OR 'Unilateral Cryptorchidism' OR 'Cryptorchidism, Unilateral' OR 'Abdominal Cryptorchidism' OR ' Cryptorchidism, Abdominal' OR 'Inguinal Cryptorchidism' OR 'Cryptorchidism, Inguinal' OR 'orchidopexy')

**Web of Science**

[(TS=(sperm retrieval rates) OR TS=(testicular sperm extraction) OR TS=(micro dissection testicular sperm extraction) OR TS=(Micro-TESE) OR TS=(M-TESE)) AND (TS=(Cryptorchidism) OR TS=(cryptoorchism) OR TS=(Testes, undistended) OR TS=(undistended Testes) OR TS=(Testis, undistended) OR TS=(cryptorchism) OR TS=(Cryptorchidism, Unilateral Or Bilateral) OR TS=(undistended Testis) OR TS=(Bilateral Cryptorchidism) OR TS=(Cryptorchidism, Bilateral) OR TS=(Unilateral Cryptorchidism) OR TS=(Cryptorchidism, Unilateral) OR TS=(Abdominal Cryptorchidism) OR TS=(Cryptorchidism, Abdominal) OR TS=(Inguinal Cryptorchidism) OR TS=(Cryptorchidism, Inguinal) OR TS=(orchiopexy))](https://webofscience.clarivate.cn/wos/alldb/summary/1317f4e7-dbc8-4194-8cf6-a0e3b8f315d0-010d016063/relevance/1)

**PUBMED**

((sperm retrieval rates) OR (testicular sperm extraction) OR(micro dissection testicular sperm extraction) OR (Micro-TESE) OR(M-TESE)) AND ((Cryptorchidism) OR (Cryptorchism) OR (Testes, Undescended) OR(Undescended Testes) OR(Testis, Undescended) OR(cryptorchid) OR(Cryptorchidism, Unilateral Or Bilateral) OR(Undescended Testis) OR(Bilateral Cryptorchidism) OR(Cryptorchidism, Bilateral) OR(Unilateral Cryptorchidism) OR(Cryptorchidism, Unilateral) OR(Abdominal Cryptorchidism) OR(Cryptorchidism, Abdominal) OR(Inguinal Cryptorchidism) OR(Cryptorchidism, Inguinal) OR(orchidopexy

))
