# Supplementary material for: Microdissection testicular sperm extraction outcomes in azoospermic patients post-orchidopexy surgery: A systematic review and meta-analysis
Source: PLoS One. 2024 Nov 15;19(11):e0313866. doi: 10.1371/journal.pone.0313866 (PMC11567534; doi:10.1371/journal.pone.0313866)

Funnel plots for sperm retrieval rates (SRR)


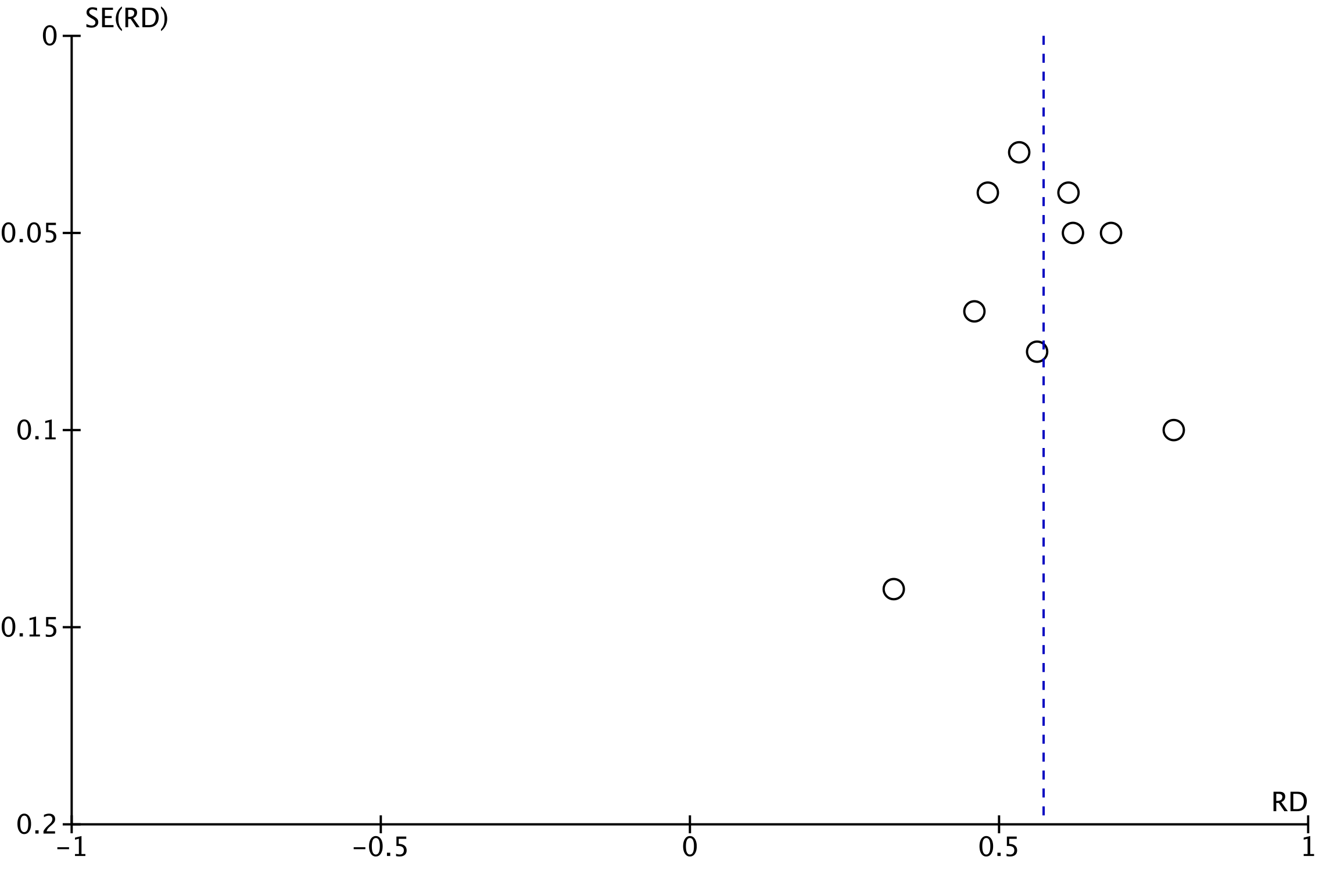


Funnel plots for Age at m-TESE


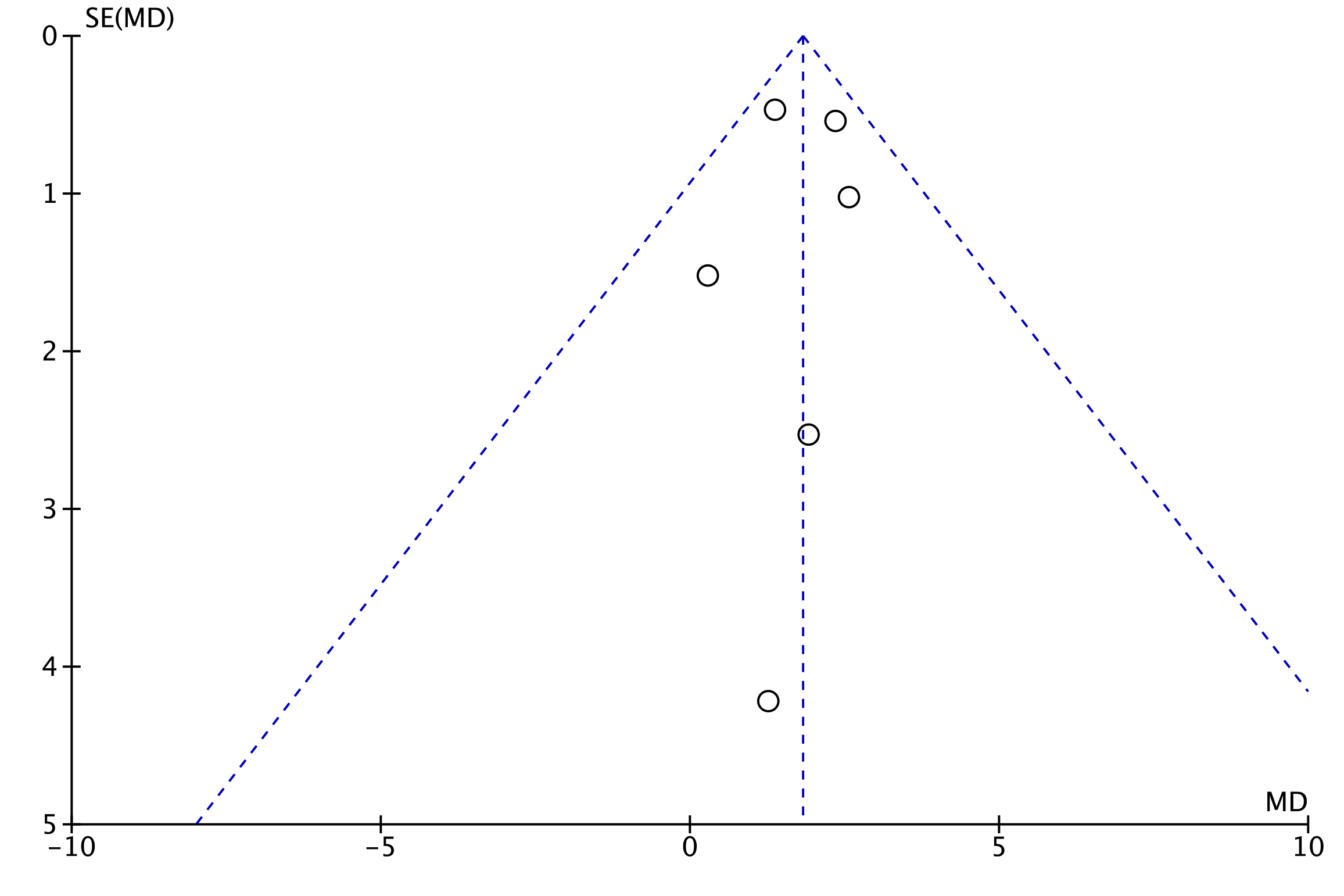


Funnel plots for Age at orchidopexy


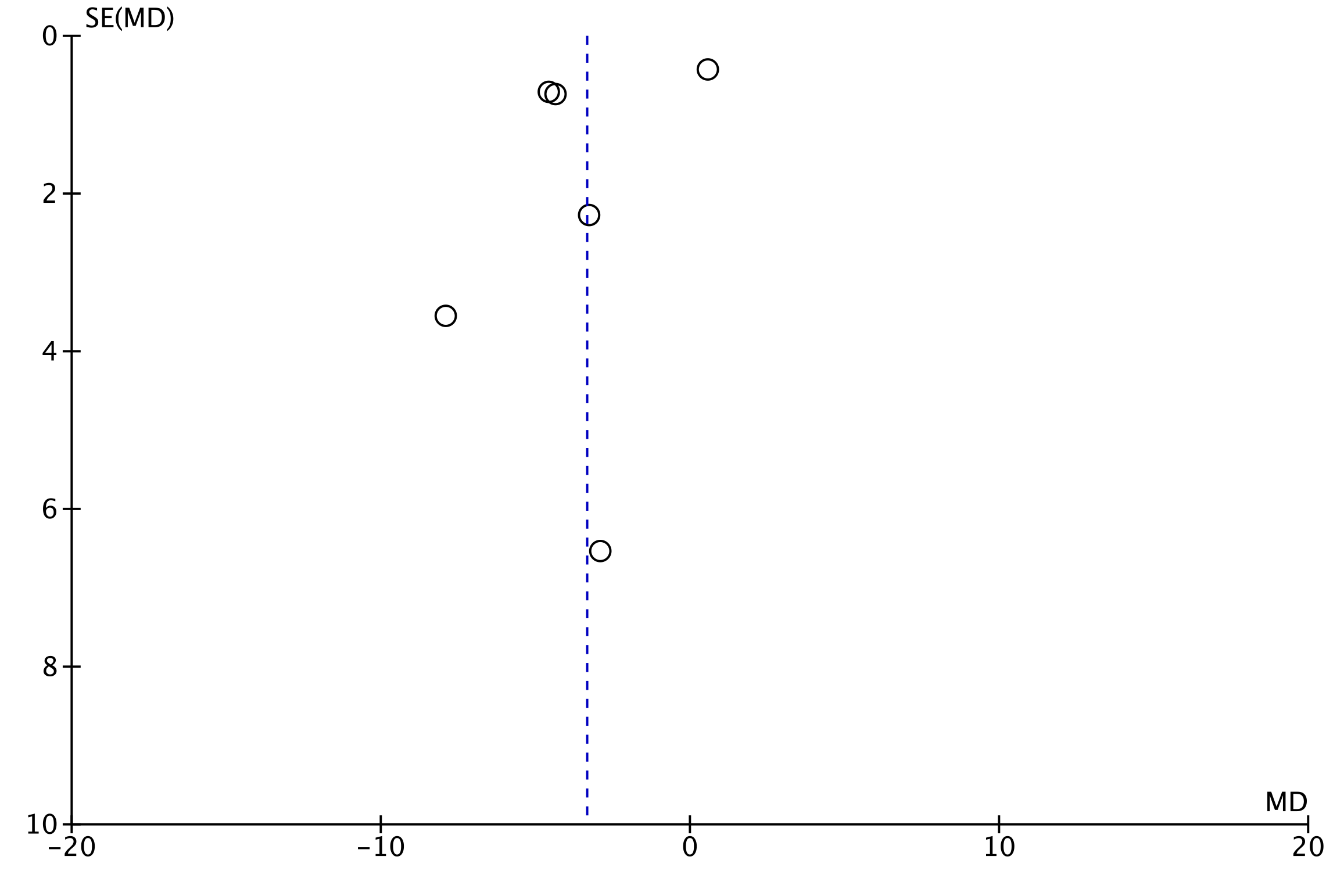


Funnel plots for Interval from orchidopexy to m-TESE
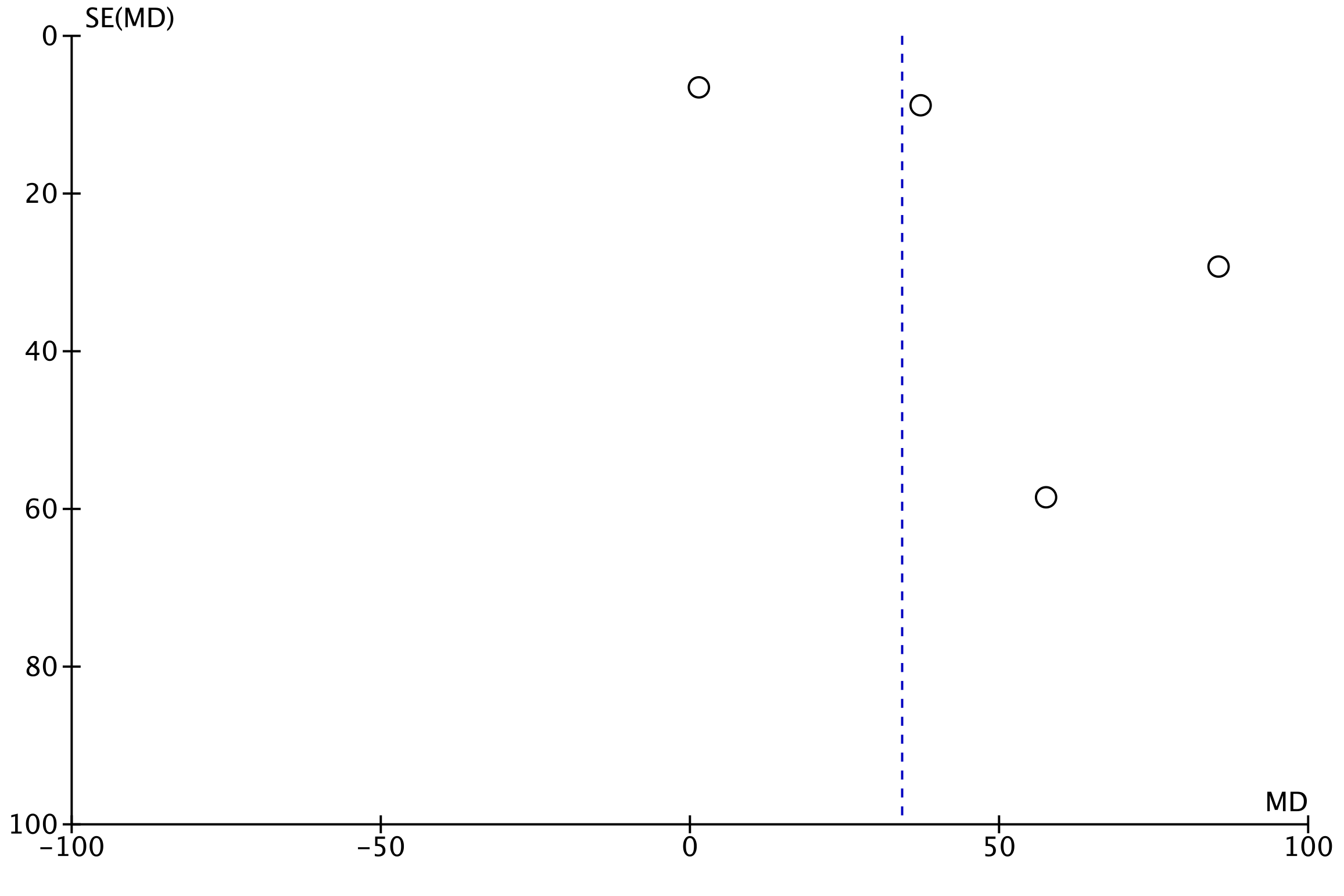


Funnel plots for Unilateral or Bilateral cryptorchidism
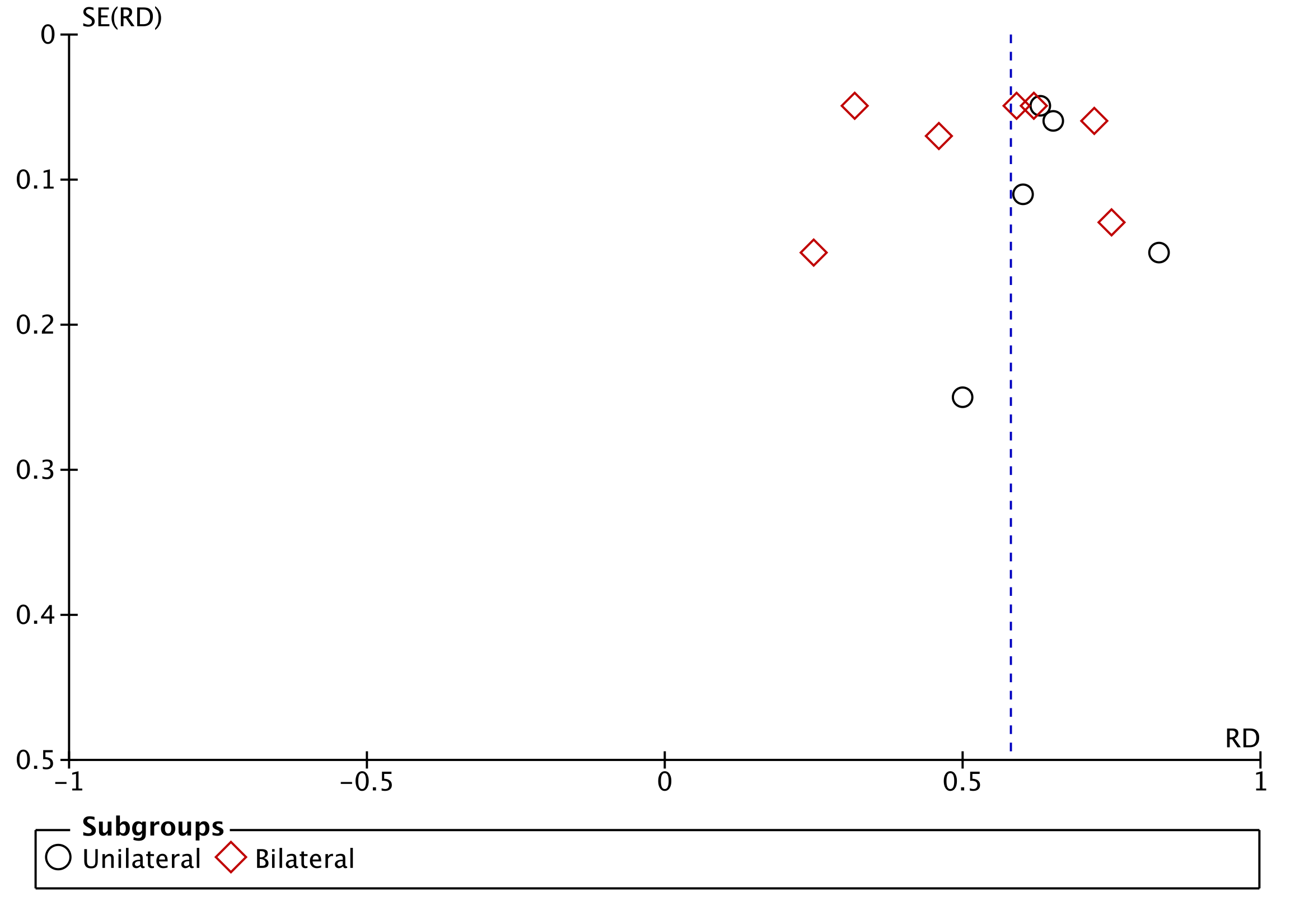


Funnel plots for Location of Undescended Testes
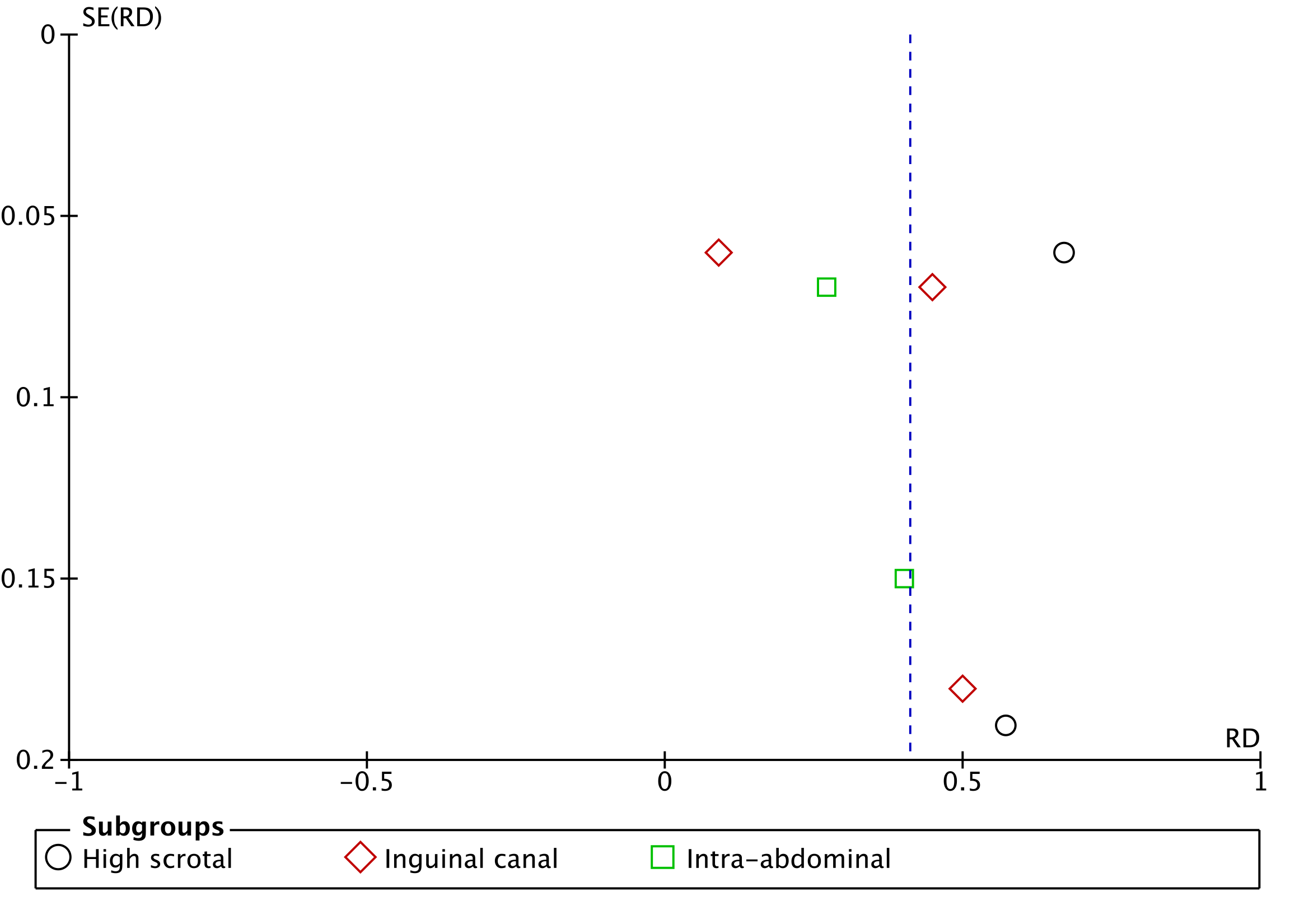


Funnel plots for testosterone (T)
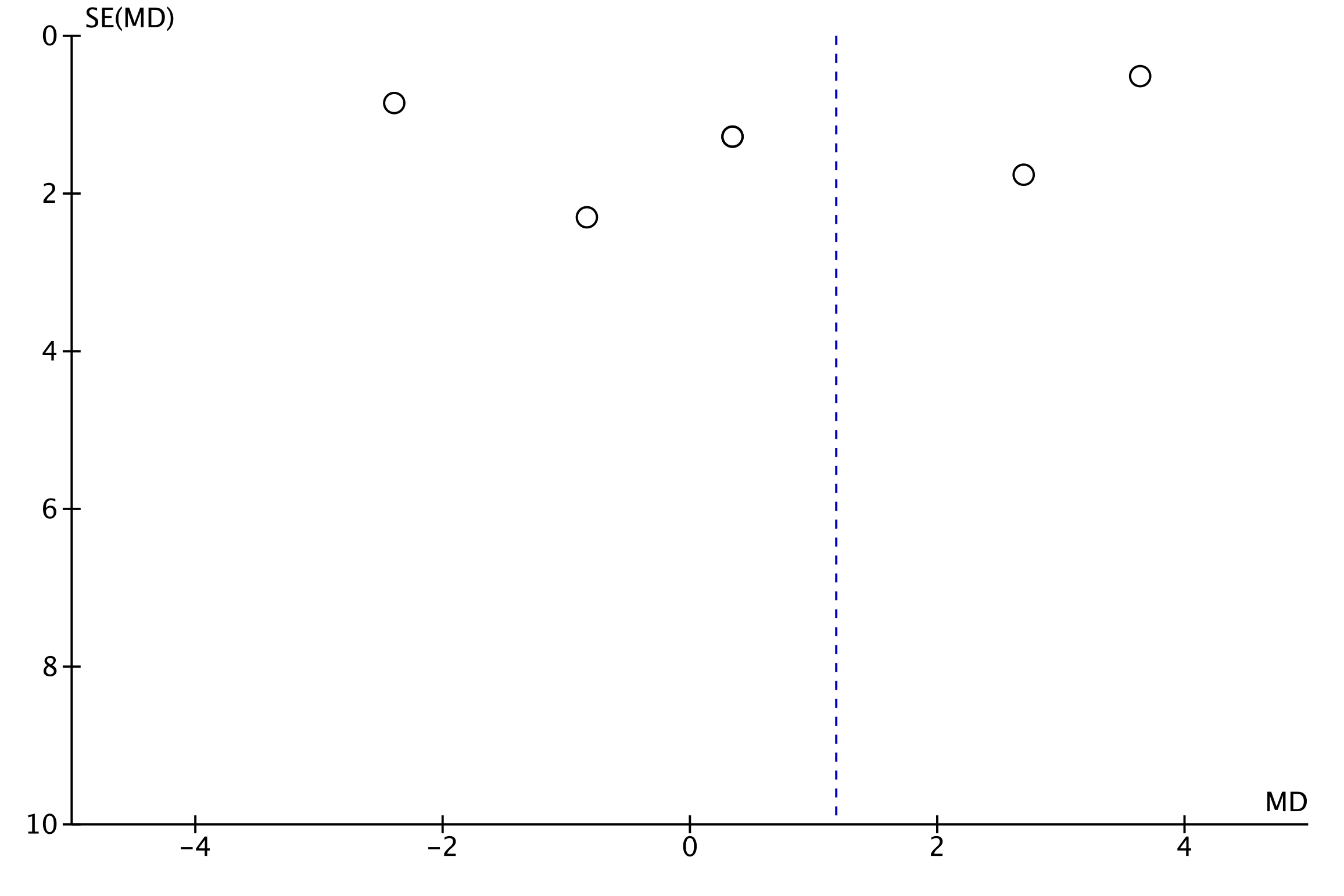


Funnel plots for follicle-stimulating hormone (FSH)
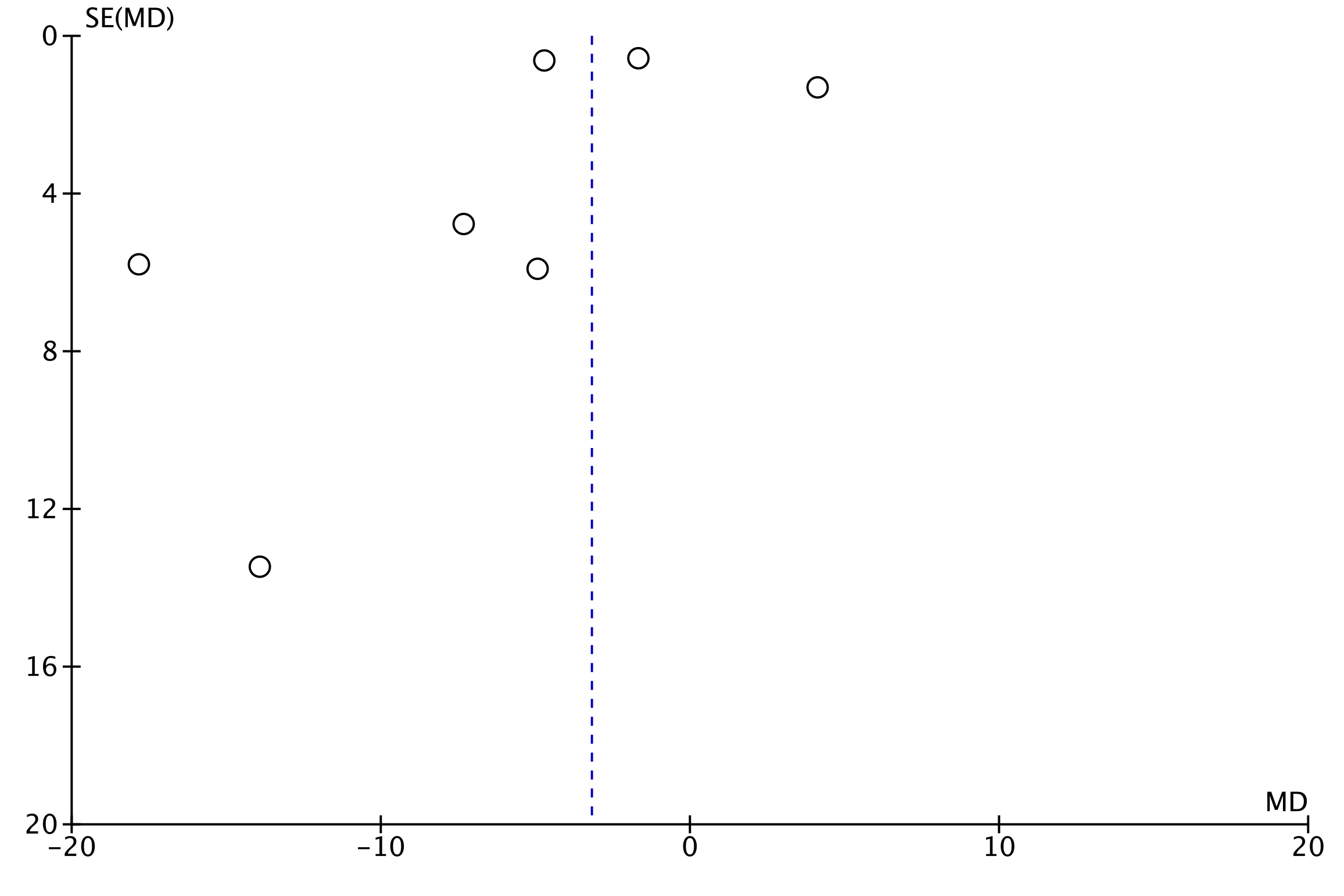


Funnel plots for luteinizing hormone (LH)
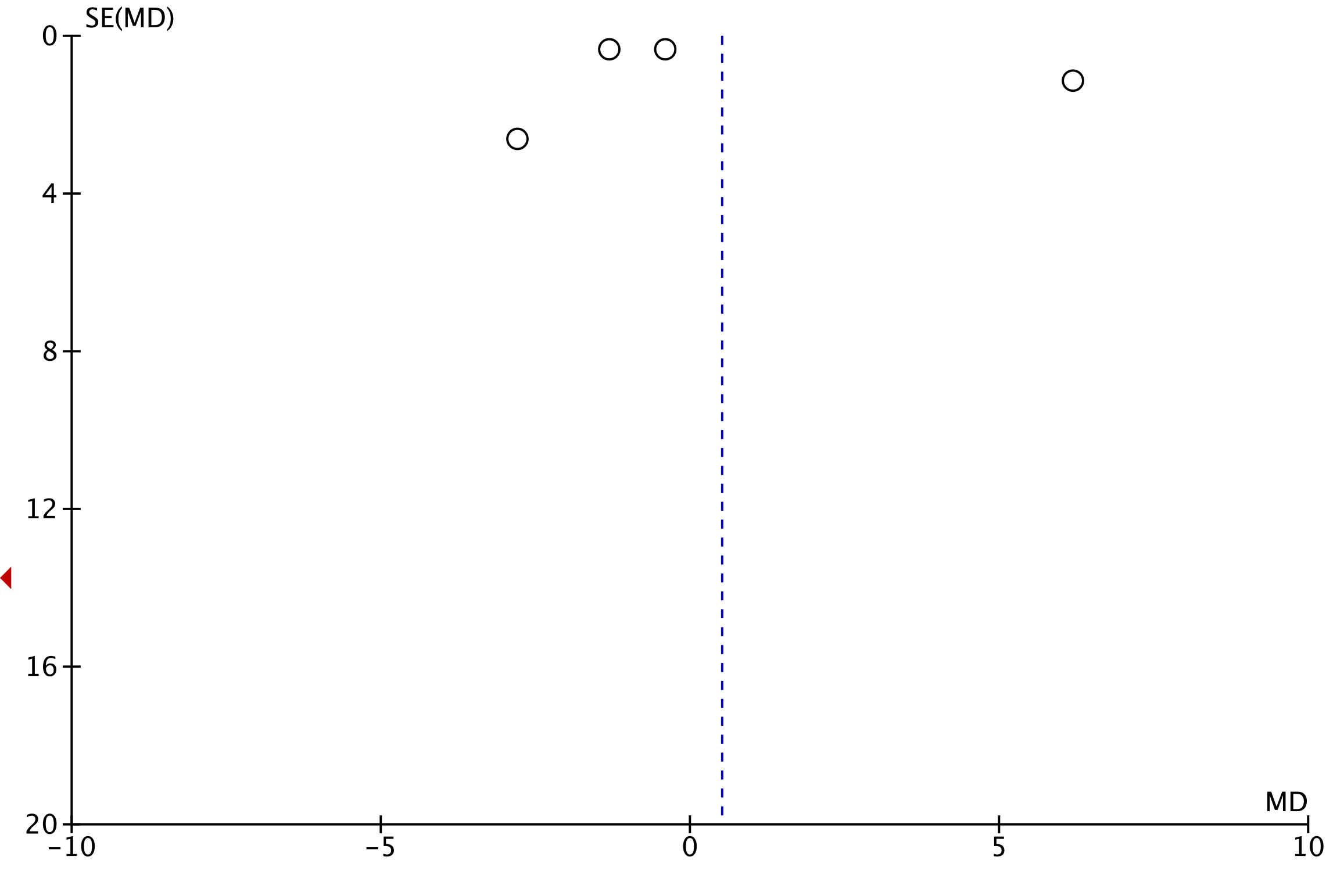


Funnel plots for testicular volume (TV)
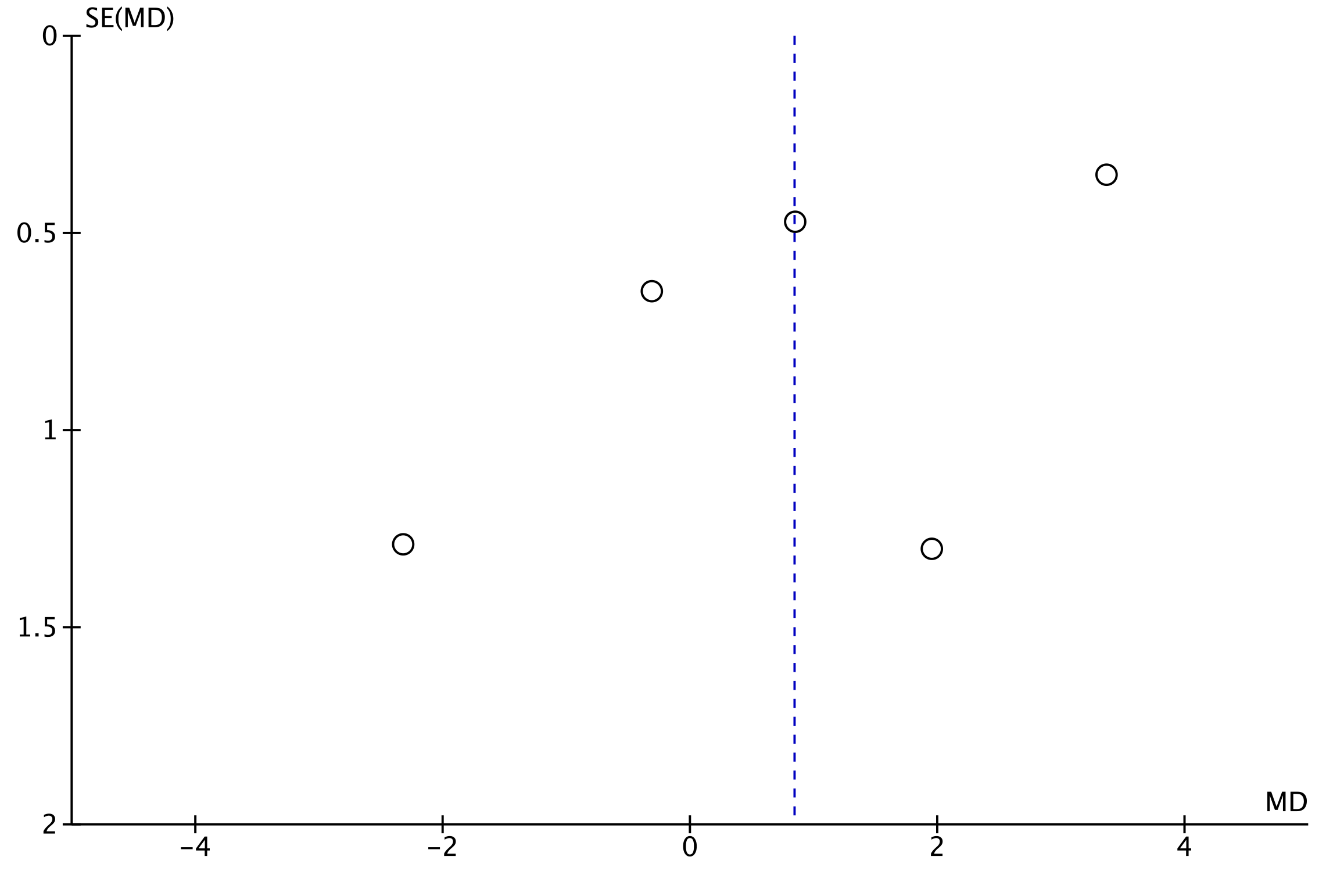

Supplement: S6 File — (DOC) [file pone.0313866.s006.doc]
